# Deciphering transcriptome complexity via long‐read sequencing

**DOI:** 10.1002/imt2.70171

**Published:** 2026-10-04

**Authors:** Chuwen Xu, Jia Li, Chenxi Yin, Keying Li, Tian Wang, Shiwen Gao, Yue Yu, Cheng Chang, Ye Wang, Guoqing Tong, Liang Gong, Wen Hu, Guoliang Chai, Yilai Han, Qian Qin, Suoqin Jin, Xiufen Zou, Bo Li, Tianyuan Zhang, Ana Conesa, Yunhao Wang, Dingjie Wang

**Affiliations:** ^1^ School of Mathematics and Statistics Wuhan University Wuhan China; ^2^ Department of Reproductive Medicine The First Affiliated Hospital of Xi'an Jiaotong University Xi'an China; ^3^ State Key Laboratory of Medical Proteomics, National Center for Protein Sciences (Beijing) Research Unit of Proteomics‐driven Cancer Precision Medicine (Chinese Academy of Medical Sciences) Beijing China; ^4^ The Conservation of Endangered Wildlife Key Laboratory of Sichuan Province Chengdu Research Base of Giant Panda Breeding Chengdu China; ^5^ Department of Urology of The First Affiliated Hospital & Liangzhu Laboratory Zhejiang University School of Medicine Hangzhou China; ^6^ Brain Cognition and Brain Disease Institute, Shenzhen Institute of Advanced Technology Chinese Academy of Sciences Shenzhen China; ^7^ Chinese Institutes for Medical Research (CIMR) Beijing China; ^8^ Department of Neurology Xuanwu Hospital Capital Medical University, National Center for Neurological Disorders Beijing China; ^9^ Beijing Municipal Geriatric Medical Research Center Beijing China; ^10^ School of Artificial Intelligence Wuhan University Wuhan China; ^11^ Stanley and Judith Frankel Institute for Heart and Brain Health University of Michigan Medical Center Ann Arbor Michigan USA; ^12^ Benagen Institute Wuhan China; ^13^ Institute for Integrative Systems Biology Spanish National Research Council Paterna Spain; ^14^ Institute for Math & AI Wuhan University Wuhan China

**Keywords:** alternative splicing, isoform resolution, long‐read sequencing, nanopore sequencing, transcript identification and quantification, transcriptome, transposable element

## Abstract

Transcriptomics is moving beyond gene‐level quantification toward isoform‐resolved interrogation of alternative splicing, transcript structural variation, and repeat‐derived transcription. Yet short‐read sequencing remains intrinsically limited in accurately reconstructing full‐length transcripts and resolving complex repetitive regions, including transposable elements. Recent advances in long‐read sequencing, exemplified by Pacific Biosciences (PacBio) and Oxford Nanopore Technologies (ONT), offer a transformative opportunity to directly observe complete RNA molecules and thereby overcome these bottlenecks. However, practical adoption is hindered by demanding library construction and the need to process noisy, fast‐evolving long‐read data, and the field still lacks a unified resource that guides researchers through the entire experimental and analytical workflow. This review fills that gap by providing a concise, end‐to‐end, and implementation‐oriented roadmap for long‐read transcriptomics. We distill the key decisions from platform and library strategy selection to core computational processing and downstream interpretation, and we summarize emerging frontiers and best‐practice recommendations. By offering a reusable framework and practical checklists, this guide empowers a broader community to exploit long reads for standardized, reproducible, isoform‐level discovery at unprecedented resolution.

## Highlights

- Long‐read sequencing enables isoform‐resolved characterization of transcriptome complexity by directly resolving full‐length transcript structures and linked regulatory features.
- The choice of sequencing platform and library preparation strategies critically influences transcript completeness, accuracy, and biological interpretation.
- Standardized computational workflows are needed for reproducible and scalable long‐read transcriptomics studies.
- Single‐cell, spatial, direct RNA sequencing, and multi‐omics approaches expand long‐read omics frontiers.

## Graphical abstract

Long‐read sequencing is transforming transcriptomics from gene‐level quantification to isoform‐resolved interpretation by directly resolving full‐length transcript structures, transcription in repetitive regions, and linked molecular features. This review provides an end‐to‐end roadmap covering sequencing platforms, library preparation strategies, computational workflows, the analysis of transcriptome complexity, and downstream analyses. It further highlights current challenges and emerging frontiers, including single‐cell and spatial transcriptomics, direct RNA modification detection, and integrated multi‐omics approaches.

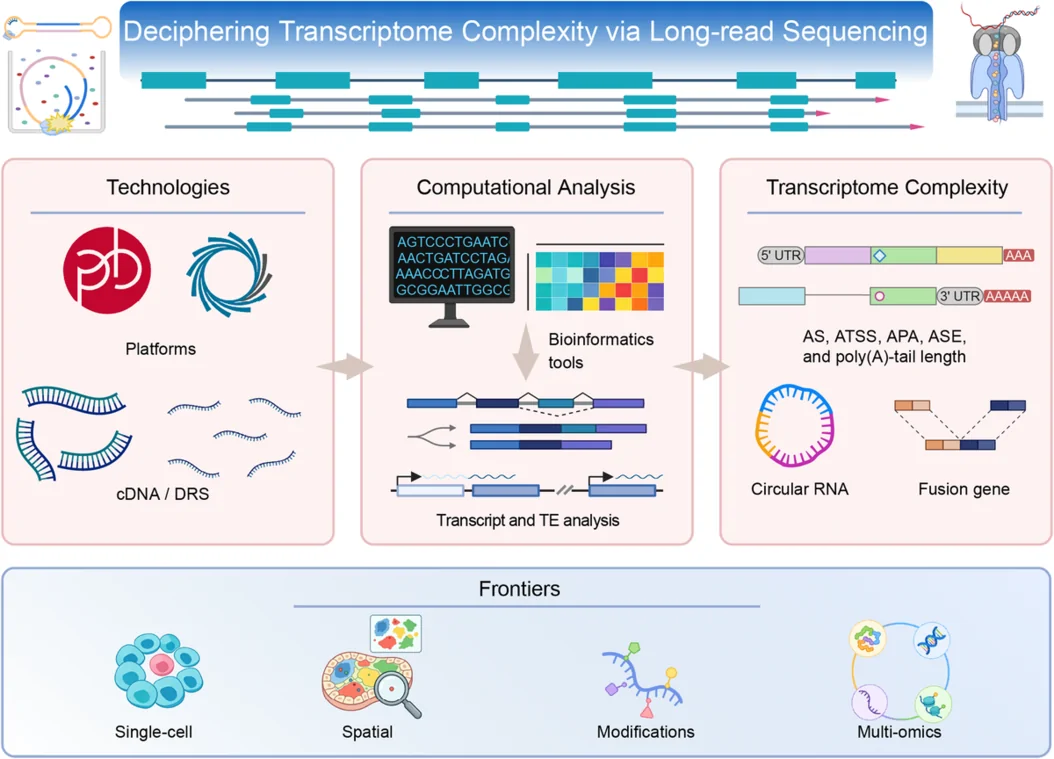

## INTRODUCTION

Transcriptomics is undergoing a major shift from gene‐level measurement to transcript‐level interpretation. For many years, short‐read RNA sequencing (RNA‐seq) has served as the gold standard for transcriptome analysis, enabling robust and scalable quantification of gene expression across conditions [1, 2]. However, growing evidence shows that many key biological and disease‐related processes are more directly reflected at the transcript level. Alternative splicing, alternative transcription start site (ATSS), alternative polyadenylation (APA), gene fusion, transposable element (TE)‐associated transcription, and allele‐specific expression (ASE) often provide the most immediate link between regulatory variation and phenotypic diversity [3, 4, 5, 6]. In cancer, pathogenic effects may arise from specific splice variants or abnormal fusions rather than total gene expression [7, 8]. In complex systems such as the brain and immune system, differences in isoform usage often distinguish cell types and states more precisely than gene‐level signals [9, 10].

This change in focus also highlights the limitations of second‐generation sequencing (often referred to as next‐generation sequencing, NGS) in terms of read length. Because NGS short reads capture only transcript fragments, full‐length transcript reconstruction often relies on assembly and statistical inference, which can be challenging when isoforms share exons and splicing junctions are combinatorially used, or transcription occurs in repetitive regions [11, 12, 13, 14]. As a result, short‐read approaches remain highly effective for gene‐level quantification, but are inherently constrained when the goal is to resolve transcript structures and isoform usage. More importantly, some transcriptomic features are not merely difficult to infer from short reads, but are fundamentally inaccessible or only indirectly observable, including RNA modifications, TE‐derived transcripts, complex fusion structures, and molecularly linked multiomic information [14, 15, 16, 17].

Long‐read sequencing (LRS) offers a straightforward approach to bridge this gap (Figure 1A). By generating reads that span several kilobases or even full‐length RNA molecules, LRS platforms make it possible to observe exon connectivity, transcript boundaries, and complex transcript structures within single molecules [13, 18]. This greatly reduces the ambiguity that limits short‐read analyses and provides a more direct foundation for transcript discovery, annotation improvement, and isoform‐level quantification [19, 20]. Beyond improving analyses that are already possible with short reads, LRS also enables fundamentally new biological measurements: direct RNA sequencing (DRS) can preserve native RNA molecules and provide access to RNA modifications [14, 21]; long reads can disambiguate transcripts arising from repetitive elements and improve TE‐derived transcript quantification [17, 22]; single‐molecule reads can reveal fusion transcript architecture and isoform context [23, 24]; and emerging long‐read multiomic strategies can link transcript identity with additional molecular features in the same molecule or cell [15, 25, 26].

**FIGURE 1:**
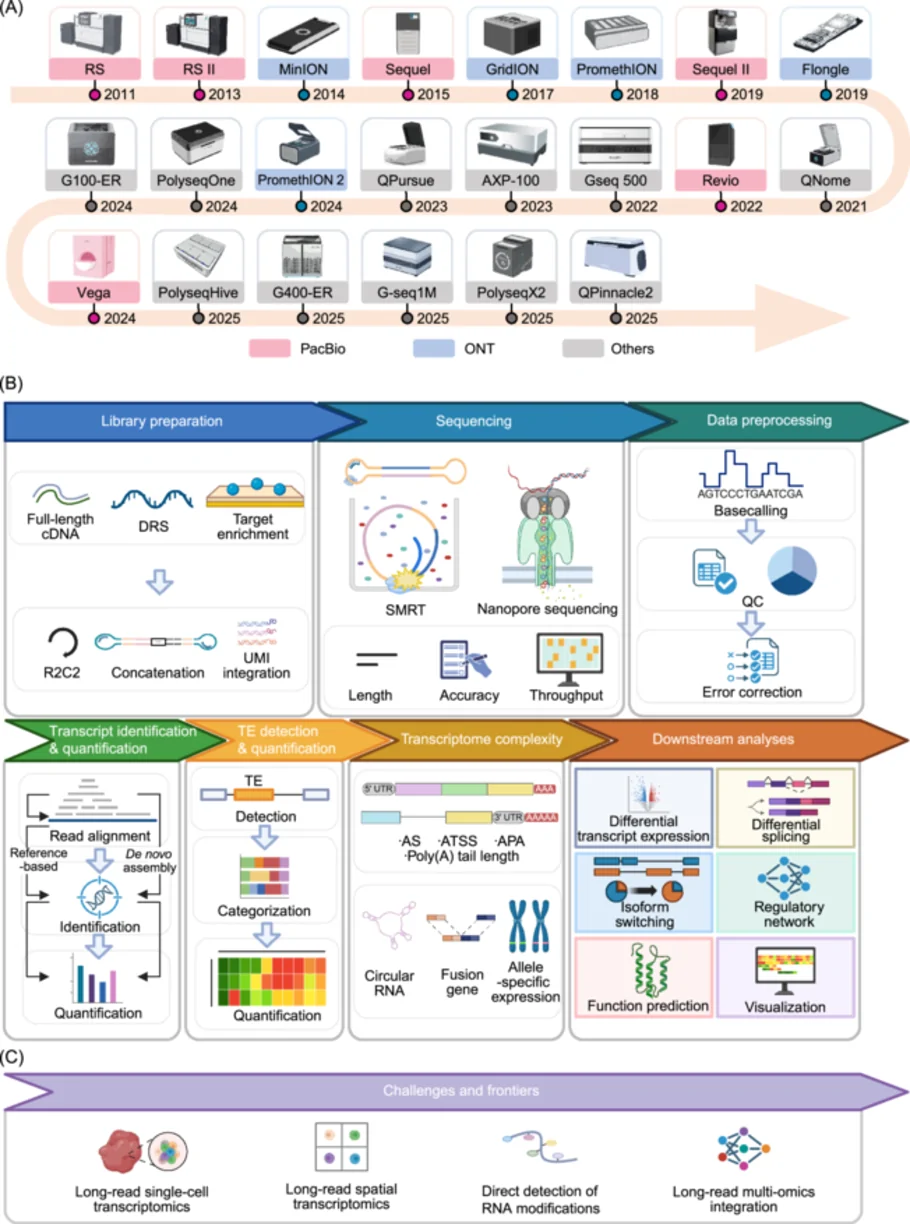
A panoramic overview of long‐read transcriptomics workflow. (A) Developmental timeline of LRS platforms from PacBio (pink), ONT (blue), and others (gray). (B) Overview of end‐to‐end workflow for LRS‐based transcriptomics studies. (C) Frontiers of long‐read transcriptomics toward single‐cell, spatial, direct RNA sequencing, and multi‐omics integration applications. AS, alternative splicing; ATSS, alternative transcription start site; APA, alternative polyadenylation; PacBio, Pacific Biosciences; cDNA, complementary DNA; DRS, direct RNA sequencing; ONT, Oxford Nanopore Technologies; R2C2, Rolling Circle to Concatemeric Consensus; UMI, unique molecular identifier; SMRT, single‐molecule real‐time; QC, quality control; TE, transposable element; UTR, untranslated region. Created in BioRender. Li, J. (2026) https://BioRender.com/a3awsvn.

Technologies such as Pacific Biosciences (PacBio) and Oxford Nanopore Technologies (ONT) have driven the rapid development of this field through full‐length complementary DNA (cDNA) sequencing and DRS strategies [27, 28]. Meanwhile, several nanopore‐based LRS platforms (e.g., QitanTech, CycloneSEQ, and Axbio) are expanding the technological landscape and accelerating local deployment of long‐read omics [29, 30, 31, 32]. Although these emerging platforms are still being evaluated in terms of performance and best‐practice workflows, they are broadening instrument choice, improving accessibility, and creating new opportunities for long‐read transcriptome research. With continuing improvements in length, accuracy, throughput, and cost, long‐read transcriptomics is moving from a specialized approach for transcript discovery toward a practical tool for studying transcriptome complexity across diverse biological contexts [33, 34, 35, 36].

Despite its great promise, long‐read transcriptomics remains technically complex and lacks standardized workflows. Its successful application requires both rigorous experimental design and careful computational analysis. Different platforms and library construction strategies, such as full‐length cDNA versus DRS, amplification‐based versus amplification‐free protocols, and targeted versus whole‐transcriptome workflows, can introduce distinct biases in read length, coverage, and error profile that propagate to affect transcript discovery [37, 38]. Moreover, long‐read data have distinct data structures and error profiles compared with short‐read data and require dedicated computational workflows for signal processing/basecalling, quality control, error correction, alignment, transcript reconstruction, annotation, and quantification. Downstream workflows for alternative splicing, fusion transcripts, ASE, circular RNAs, RNA modifications, and repeat‐derived transcription add further complexity [1, 39]. Although many tools and several benchmarking studies have emerged in recent years [40, 41, 42, 43, 44], the field is still evolving rapidly, and performance trade‐offs remain insufficiently characterized. Importantly, despite the enormous potential of long‐read transcriptomics, there is still a lack of comprehensive resources that can guide researchers in a systematic way through the full workflow from experimental design to data interpretation.

In this review, we provide a practical end‐to‐end guide to long‐read transcriptomics. We highlight the value of long‐read RNA‐seq for resolving transcriptome complexity and reveal molecular features that are difficult or impossible to access with short reads alone. We provide an integrated overview of long‐read transcriptomics, covering key technologies, study design, library preparation, data processing, transcript identification, quantification, and downstream analysis (Figure 1B), while also discussing current challenges and future directions (Figure 1C). By bringing these aspects together within a unified framework, this review is intended to help researchers more effectively design, perform, and interpret long‐read transcriptome studies.

## TECHNOLOGIES: PLATFORMS, PROTOCOLS, AND PERFORMANCE CHARACTERISTICS

### Long‐read sequencing platforms

In addition to the two dominant LRS technologies (PacBio and ONT) that were commercialized in the 2010s, several new nanopore‐based LRS platforms have emerged in recent years [37, 45]. We first provide an overview of PacBio and ONT, followed by a brief discussion of these emerging platforms (Table S1).

### Pacific Biosciences

As the earliest commercialized LRS technology, PacBio single‐molecule real‐time (SMRT) sequencing adopts a sequencing‐by‐synthesis principle, enabling polymerase‐driven synthesis reactions within nanoscale zero‐mode waveguides (ZMWs) [46, 47] (Figure 2A). PacBio employs a SMRTbell library, a circular DNA template to allow a single polymerase to traverse the same DNA insert multiple times, thereby improving consensus accuracy. During nucleotide incorporation, fluorescent signals are recorded alongside interpulse duration (IPD), which provides additional kinetic information and can help infer DNA base modifications such as N^6^‐methyladenine [48, 49].

**FIGURE 2:**
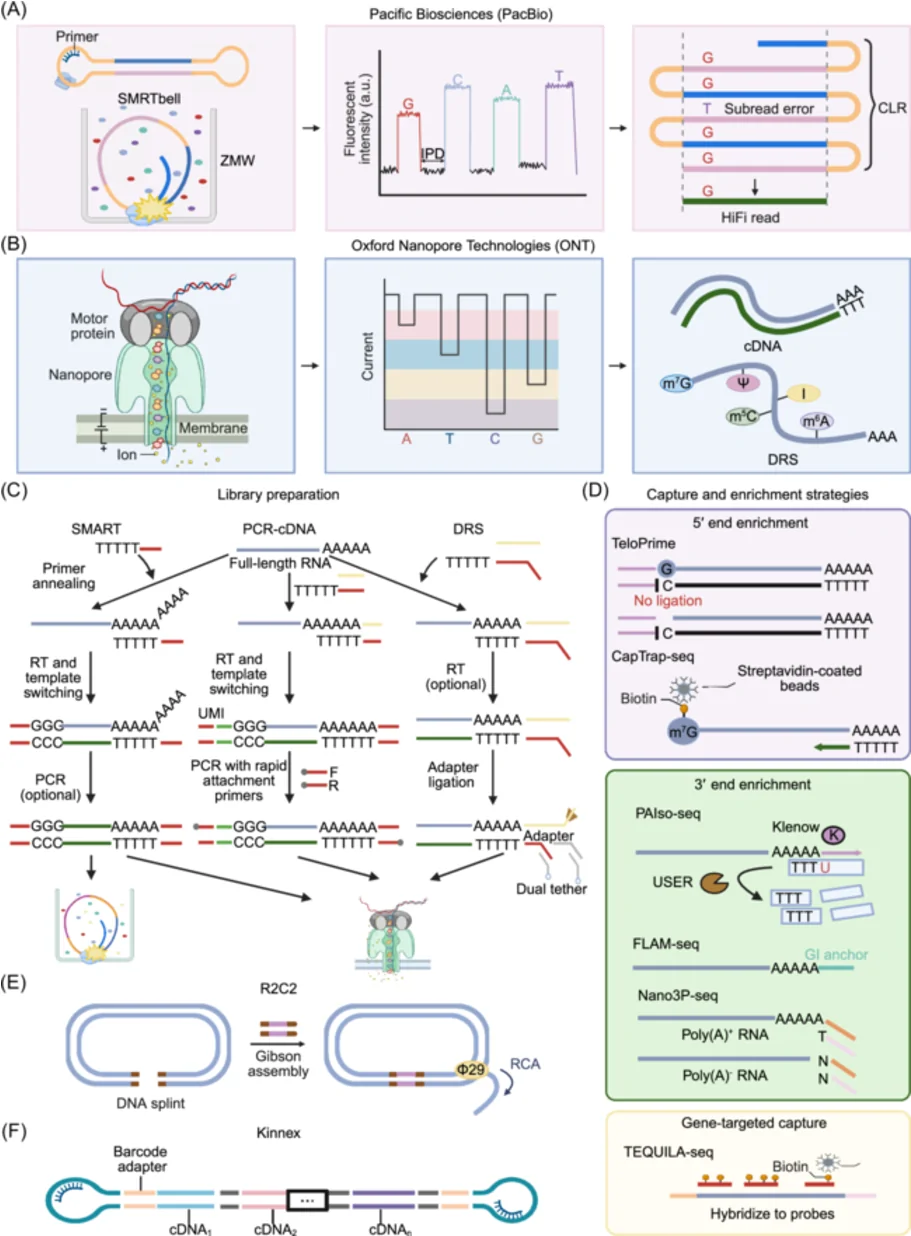
Overview of LRS platforms and principles, and library preparation and enrichment strategies for transcriptomics studies. (A) For PacBio sequencing, a SMRTbell circular template is loaded into a ZMW and sequenced by a polymerase; fluorescence pulses and IPD signals are recorded; and repeated passes produce multiple subreads to yield a consensus HiFi read. (B) For ONT sequencing, a motor protein controls strand translocation through a nanopore; the pore generates an ionic‐current “squiggle” determined by local sequence context; and the squiggle is basecalled into sequence, while RNA base modifications can be retained in the raw signal when DRS is performed. (C) Three major workflows for long‐read RNA‐seq library preparation: SMART, ONT's PCR‐cDNA, and DRS. (D) Representative capture and enrichment strategies: 5'‐end enrichment (e.g., CapTrap‐seq), 3'‐end enrichment approaches (e.g., PAIso‐seq), and gene‐targeted capture (TEQUILA‐seq). (E) R2C2: rolling‐circle amplification creates repeated units that are sequenced and assembled into a higher‐accuracy consensus sequence. (F) Kinnex: concatenation of multiple cDNA fragments into a single long template increases sequencing throughput. cDNA, complementary DNA; CLR, continuous long reads; DRS, direct RNA sequencing; FLAM‑seq, full‑length mRNA sequencing; IPD, interpulse duration; ONT, Oxford Nanopore Technologies; PacBio, Pacific Biosciences; PAIso‑seq, poly(A) inclusive RNA isoform sequencing; PCR, polymerase chain reaction; RT, reverse transcription; SMRT, single‑molecule real‑time; SMART, switching mechanism at the 5' end of RNA template; R2C2, rolling circle to concatemeric consensus; RCA, rolling circle amplification; UMI, unique molecular identifier; ZMW, zero‑mode waveguide. Created in BioRender. Li, J. (2026) https://BioRender.com/bjr2lnu.

Given that polymerase lifetime is finite and the SMRTbell library is circular, shorter DNA inserts yield more passes and thus higher consensus accuracy [28]. PacBio offers two core modes to meet diverse research needs. Continuous long reads (CLR), the initial mode, delivers ultra‐long read length but suffers from a high error rate. In contrast, high‐fidelity (HiFi) reads, generated using circular consensus sequencing (CCS), take short DNA templates as input and thus offer high accuracy. PacBio established Iso‐Seq as its flagship solution for full‐length transcriptome studies [18]. Conceptually, Iso‐Seq directly leverages the HiFi mode: by converting reverse‐transcribed full‐length cDNA into a SMRTbell library and subjecting it to multiple passes of CCS, each transcript generates a highly accurate consensus read.

### Oxford nanopore technologies

Nanopore sequencing, pioneered by ONT, detects single‐stranded DNA or RNA molecules by monitoring ionic‐current disruptions as they translocate through a nanometer‐scale pore [13, 50, 51] (Figure 2B). Two key components underpin nanopore sequencing: the pore protein (i.e., nanopore) that senses electrical current changes, and the motor protein that unwinds double‐stranded molecules and controls the translocation speed of single‐stranded DNA/RNA molecules through the nanopore [52]. Because the signal is primarily determined by a short sequence context (*i.e*., k‐mer) residing in the constriction zone, each step of strand movement produces a characteristic squiggle transition [53]. These squiggle signals are translated into bases by probabilistic and deep‐learning basecallers (*e.g*., Guppy (https://nanoporetech.com/zh/software/other/guppy/, now upgraded to Dorado), Dorado (https://github.com/nanoporetech/dorado/), and Bonito (https://github.com/nanoporetech/bonito/)) trained for specific nanopore types and chemistries [53, 54].

In transcriptomics applications, ONT employs two major strategies: cDNA sequencing and DRS [13, 27]. While DRS enables sequencing of native RNA molecules and direct detection of RNA modifications such as N^6^‐methyladenosine (m^6^A), its adoption remains limited in practice due to higher RNA amount requirements, lower throughput, and increased susceptibility to 5′ end truncation and RNA degradation. cDNA sequencing can be further divided into direct cDNA sequencing and polymerase chain reaction (PCR)‐amplified cDNA sequencing. PCR‐amplified cDNA sequencing has become the dominant approach in most studies, offering greater sensitivity, robustness, and compatibility with low‐input samples. Therefore, we focus primarily on PCR‐cDNA workflows for data analysis in this review.

### Other long‐read sequencing platforms

Beyond PacBio and ONT, several emerging LRS systems have been developed [29, 32, 55, 56]. While these platforms may broaden access and diversify the commercial supply of sequencing instruments, many of their performance metrics (e.g., sequencing length, accuracy, and throughput) are still reported primarily by manufacturers and derived from early‐stage studies [57, 58].

QitanTech (https://www.qitantech.com), Polyseq (https://www.polyseq.com), and CycloneSEQ (https://www.cyclone-seq.com) [31] share the same fundamental principle as ONT but employ different motor and pore proteins. Axbio (https://www.axbio.cn) and Geneus (http://www.geneus-tech.com) are also based on nanopore technology, yet they differ in pore protein design, signal detection system, and basecalling algorithm compared with ONT. Both platforms integrate sequencing‐by‐synthesis with electrical detection, rather than optical readout. DNA polymerase and a nanopore are immobilized on the surface of a sequencing chip. As the DNA template is replicated, modified nucleotides carrying unique electroactive tags are incorporated one by one into the growing complementary strand. Upon incorporation, each tag passes through the nanopore, generating a characteristic change in an electrical property, which is detected in real‐time by highly sensitive sensors embedded in the chip. These specific electrical signals are interpreted to determine the corresponding base.

Roche's Sequencing by Expansion (SBX) uses a biochemical conversion process to encode nucleic acid sequences into Xpandomer molecules to amplify sequence signals and overcome intrinsic signal‐to‐noise limitations [59]. Element Biosciences (https://www.elementbiosciences.com) offers a distinct long‐read approach called LoopSeq [60]. LoopSeq fragments and barcodes long DNA molecules into shorter pieces, which are then sequenced on a short‐read platform, followed by computational reassembly of the original long DNA molecules.

### Library preparation strategies

LRS platforms lay the technical foundation for deciphering full‐length transcriptomes, but library preparation is the pivotal factor that directly modulates the completeness and efficiency of full‐length transcript capture. The overarching objective is to faithfully preserve the structural integrity of RNA molecules from the 5′ cap to the 3′ poly(A) tail (Table S2) [2].

For full‐length transcriptome sequencing, an end‐to‐end workflow for RNA preservation and handling should be established, including rapid stabilization or freezing, controlled cold‐chain transport, and RNase‐minimizing procedures. For instance, bead‐based extraction and cleanup methods (*e.g*., magnetic nanoparticles) can improve sample integrity and reproducibility relative to phenol‐chloroform workflows [61]. Prior to library preparation, it is advisable to assess RNA quantity using Qubit, quality using Nanodrop, and integrity using TapeStation (*e.g*., RNA integrity number (RIN) and size distributions) [62]. With RNA preservation and quality control established, the next central decision is the choice of library preparation strategies: whether to convert RNA into full‐length cDNA followed by DNA sequencing or to sequence the native RNA molecule directly (Figure 2C).

### Full‐length cDNA generation

Converting RNA into full‐length cDNA is the most widely adopted and robust framework for long‐read transcriptomics studies. The switching mechanism at the 5′ end of RNA template (SMART) technology is widely used for full‐length cDNA preparation [63, 64]. The process begins with reverse transcription (RT) primed from the 3' poly(A) tail. Upon reaching the 5' cap, a specialized reverse transcriptase (e.g., Moloney murine leukemia virus (MMLV), SuperScript II, Maxima H Minus) adds non‐templated deoxycytidines via its terminal transferase activity [65]. A specially designed template‐switching oligonucleotide (TSO) bearing a 3' deoxyguanosine or locked nucleic acid‐modified guanylate anneals to this overhang, allowing the reverse transcriptase to switch templates and continue synthesis to the end of the TSO [64]. Because template switching occurs efficiently only at authentic 5' ends, prematurely terminated cDNAs are selectively excluded from subsequent amplification, inherently enriching the library for full‐length clones [66, 67].

Compared with conventional full‐length cDNA synthesis methods [68] and earlier cap‐dependent technologies (*e.g*., oligo‐capping [69], CAPture [70], and CAP‐trapper [71]), SMART is a more streamlined approach that requires fewer enzymatic steps and circumvents cap degradation risks. SMART has been progressively optimized for low‐input and even single‐cell applications by Rickard Sandberg's group [66, 67, 72]. Commercial kits from Takara/Clontech for full‐length cDNA preparation are built upon this core SMART principle [64].

PacBio Iso‐Seq adopts the SMART principle to generate full‐length cDNA [63] (Figure 2C). The amplified double‐stranded cDNA is then converted into a SMRTbell library, a circular DNA template with hairpin adapters ligated to both ends. This conversion begins with DNA damage repair and end repair/A‐tailing steps, which prepare the blunt‐ended cDNA for adapter ligation. The prepared cDNA is then ligated to hairpin adapters. These adapters form the characteristic dumbbell‐shaped structure, where the cDNA insert becomes a circular template flanked by hairpin loops. After adapter ligation, the library is purified using AMPure PB beads to remove unligated adapters and reaction byproducts. Optional size selection may be performed using systems like BluePippin to enrich for specific fragment length fractions (e.g., 1–3 kb, 3–6 kb, >6 kb) depending on the experimental objectives and research aims. This size selection helps optimize sequencing efficiency on early PacBio platforms (e.g., RS II) [73, 74, 75]. Finally, the purified library is annealed with sequencing primers, bound with DNA polymerase, and loaded for sequencing.

ONT cDNA sequencing library preparation follows a different workflow from PacBio Iso‐Seq, as it produces a linear library suitable for nanopore sequencing. ONT offers two distinct cDNA sequencing approaches: PCR‐cDNA sequencing and direct cDNA sequencing, which differ primarily in whether PCR amplification is included. Direct cDNA sequencing without PCR minimizes amplification‐related biases (e.g., nucleotide misincorporation, short‐transcript preference) and thus improves data quality [76, 77], while PCR‐cDNA sequencing requires less RNA, facilitating effective sequencing for samples with limited materials (e.g., mammalian early embryos) [78, 79]. Of note, PCR‐cDNA sequencing is the primary method used by the community, and direct cDNA sequencing is no longer supported by ONT [42, 78]. Therefore, we focus on PCR‐cDNA library preparation below.

ONT PCR‐cDNA library preparation (Figure 2C) consists of three main steps: RT and strand‐switching, PCR amplification, and sequencing adapter attachment [13]. The RT step begins with the ligation of the cDNA RT adapter (CRTA) to the RNA template. The CRTA is a double‐stranded adapter with a poly(T) overhang that anneals to the very end of the poly(A) tail of the RNA strand. This design ensures that the full length of the RNA is reverse transcribed and allows estimation of poly(A) length. Subsequently, the bottom strand of the ligated CRTA is digested so that the RT primer can bind the CRTA sequence as a primer for RT. First‐strand cDNA synthesis is performed using Maxima H Minus. As the reverse transcriptase reaches the 5' cap of the RNA, its terminal transferase activity adds non‐templated deoxycytidines to the end of the newly synthesized cDNA [64]. The strand switching primer II (SSPII) base pairs to the deoxycytidine tail, allowing the reverse transcriptase to strand‐switch for synthesis of the second cDNA strand. Double‐stranded cDNA is then generated by PCR amplification using primers that contain 5' tags, which facilitate the ligase‐free attachment of rapid sequencing adapters. PCR amplification is performed using LongAmp Hot Start Taq, which offers exceptional performance with long amplicons. The final step involves rapid adapter attachment, where the rapid adapter binds to the 5' tags present on the amplified cDNA. For other LRS platforms that do not provide official cDNA sequencing library preparation kits or protocols, full‐length double‐stranded cDNA generated by SMART or other methods can be subjected to DNA sequencing library preparation. These platforms can treat the cDNA as standard DNA amplicons. Indeed, even for PacBio and ONT, once double‐stranded cDNA is obtained, it can be processed through their standard DNA sequencing workflows, independent of the dedicated cDNA library preparation kits [40, 80].

### Direct RNA sequencing

Pioneered by ONT, DRS is an LRS strategy that directly reads native RNA molecules without the need for cDNA intermediates [27, 81]. As a cornerstone technology for nanopore‐based transcriptomics and epitranscriptomics studies [50], DRS avoids the inherent loss of epigenetic information associated with cDNA conversion. Many studies established the feasibility of DRS: initial ONT research succeeded in the direct sequencing of yeast poly(A)^+^ RNA, followed by diverse RNA viruses (~3–11.5 kb) such as SARS‐CoV‐2 subgenomic RNAs (~8.4 kb) [27, 82, 83]. Subsequently, sequencing of full‐length *Escherichia coli* 16S rRNA proved the technology's ability to detect specific known modification sites, such as m^7^G and pseudouridine [84].

The canonical DRS library workflow relies on the poly(A) tail of RNA molecules: a duplex adapter with an oligo(dT) overhang is first ligated to the poly(A) tail of RNAs [85] (Figure 2C). An optional RT step can be included to stabilize RNA structure and improve sequencing yield, though the sequencing template remains strictly RNA. A sequencing adapter pre‐loaded with a motor protein is sequentially ligated. Following the removal of the free adapter using RNAClean XP beads, the prepared library is loaded for sequencing. During sequencing, the motor protein drives the RNA molecule through the nanopore in the 3'–5' direction, generating characteristic ionic current signals. Basecalling algorithms subsequently reverse the data, displaying sequencing reads in the 5'–3' direction.

Due to the 3'–5' sequencing direction, premature termination or RNA degradation can result in loss of coverage at the 5' end. Notably, DRS typically requires larger amounts of RNA (e.g., 300 ng poly(A)^+^ RNA) compared to PCR‐cDNA sequencing (e.g., 10 ng poly(A)^+^ RNA), as DRS does not involve PCR amplification [86, 87].

### Enrichment strategies: From end integrity to targeted capture

In addition to the library preparation protocols officially released by PacBio and ONT, several third‐party enrichment strategies have been developed: end‐constrained strategies to prioritize intact transcript termini [37], and gene‐targeted capture to maximize detection sensitivity for predefined transcript panels [88] (Figure 2D and Table S2). Accurate characterization of full‐length transcripts requires enrichment methods that selectively capture RNA molecules with intact 5′ caps and 3′ poly(A) tails, thereby effectively reducing degraded or prematurely terminated species.

Several techniques have been developed to enrich full‐length RNA molecules with intact 5' caps, such as TeloPrime and CapTrap‐seq. TeloPrime, commercialized by Lexogen, employs a proprietary cap‐dependent linker ligation (CDLL) mechanism in conjunction with long RT [63]. In this workflow, full‐length cDNA synthesis is initiated by oligo(dT)‐primed RT, generating a stable RNA‐cDNA hybrid [27]. The CDLL reaction then utilizes a double‐stranded adapter that base pairs atypically with the inverted guanosine of the 5' cap structure. The 5' linker is ligated only when an intact 5' cap is present and the RT has reached the 5' end of RNA, effectively excluding degraded RNAs or incomplete cDNAs.

CapTrap‐seq builds on established Cap‐trapping strategies [71, 89, 90, 91], but incorporates optimizations tailored for LRS [92, 93]. Following poly(A) enrichment and anchored oligo(T)‐primed first‐strand synthesis, the initial round of full‐length selection occurs via Cap trapping: the 5′ cap of intact RNA molecules is biotinylated, enabling streptavidin‐mediated capture of capped full‐length transcripts. An RNase treatment step then cleaves single‐stranded RNA regions, removing uncapped RNAs. A second round of selection follows, involving double‐stranded linker ligation dependent on both the 5′ cap and poly(A) tail structures, further enriching for complete cDNA molecules. Finally, PCR amplifies full‐length templates with high fidelity. By employing two consecutive rounds of selection, CapTrap‐seq effectively reduces 5′ truncation artifacts. Of note, CapTrap‐seq requires a high starting total RNA input (5 μg) and involves a relatively complex multi‐stage procedure that favors the detection of shorter RNA molecules.

Despite their superior 5' end fidelity, TeloPrime and CapTrap‐seq are constrained by their reliance on oligo(dT) priming, which can induce internal priming at A‐rich sequences within the transcript body, and cannot preserve the complete poly(A) tail at the 3′ end [94]. To address these limitations, several poly(A) tail‐focused enrichment strategies have been developed [95], such as PAIso‐seq, FLAM‐seq, and Nano3P‐seq.

PAIso‐seq circumvents conventional poly(A) magnetic bead enrichment and instead employs a dU‐modified oligo(dT) probe with Klenow fragment extension to capture poly(A) tails, thereby minimizing systematic biases against long poly(A) tails caused by the low binding efficiency of magnetic beads to long poly(A) structures [96, 97]. Subsequent USER enzyme cleavage degrades the probe, preventing pseudopriming and tail truncation [96, 98]. RT and template switching are then performed to generate cDNA. Following PCR amplification, the SMRTbell library is prepared and sequenced on the PacBio platform. At single‐molecule resolution, PAIso‐seq jointly resolves full‐length transcript isoform architecture and poly(A) tail phenotypes, including tail length dynamics and embedded non‐A residues [96, 97]. With rigorously validated subnanogram‐level RNA compatibility, it is broadly applicable across sample types, including extremely low‐input in vivo specimens [97].

FLAM‐seq is also designed to capture full‐length transcripts alongside their poly(A) tails. In this workflow, poly(A) RNAs are enzymatically tailed with a short guanosine/inosine segment, which serves as an anchoring site for oligonucleotides bearing unique molecular identifiers (UMIs) and PCR handles. RT is then performed in conjunction with a chemically modified TSO that tags the 5' ends of RNA molecules with a second PCR handle. After PCR amplification, the SMRTbell library is prepared and subjected to PacBio sequencing. FLAM‐seq's primary advantage lies in the joint measurement of the transcript body and the 3' poly(A) tail within a contiguous long read, enabling the mapping of tail‐length dynamics to specific transcript classes, UTR architectures, and APA events [99].

Distinct from CapTrap‐seq and FLAM‐seq, which are focused on poly(A)+ RNAs, Nano3P‐seq broadly enhances the recovery of transcript 3'‐end information, encompassing both polyadenylated and non‐polyadenylated transcripts [100]. By optimizing the initiation steps, it circumvents the need for 3' end‐conjugation ligation or second‐strand cDNA synthesis. Empirical evidence demonstrates that Nano3P‐seq introduces minimal library preparation bias, making it highly suitable for whole‐transcriptome composition analysis and the comparative evaluation of 3′ end processing across diverse RNA classes [101]. Notably, an alternative approach for sequencing non‐polyadenylated RNAs involves exogenous enzymes (e.g., *Escherichia coli* Poly(A) polymerase) to add poly(A) tails, followed by standard poly(A)‐dependent library preparation for LRS [102, 103].

To reduce sequencing cost and focus on specific transcripts of interest, targeted capture approaches for LRS have been developed, such as TEQUILA‐seq, ORF Capture‐Seq, RNA Capture Long Seq, multiplexed targeted sequencing, and combining long‐range RT‐PCR with ONT [104, 105, 106, 107]. TEQUILA‐seq is a cost‐effective, scalable targeted long‐read RNA‐seq strategy for candidate gene sets or specific biological pathways. It uses nicking endonuclease‐triggered isothermal strand displacement and amplification with only a limited set of initial oligonucleotides, efficiently generating customizable biotinylated probes to capture targeted transcripts. Combined with ONT for enriched cDNA sequencing, it exhibits high capture efficiency, specificity, and uniform target enrichment across gene panels of varying sizes [88]. Additionally, TEQUILA‐seq can be easily adapted into a method for sequencing all non‐polyadenylated transcripts via a simple polyadenylation process, and subsequent design of targeted probes for non‐polyadenylated transcripts of different lengths will further enhance its utility [108, 109]. For studies with constrained sequencing budgets focusing on isoform dynamics in a defined gene panel, targeted enrichment increases the on‐target read fraction, improving the detection sensitivity and quantitative robustness of rare isoforms, low‐abundance splicing events, and condition‐specific transcripts. As a key precision medicine approach, it facilitates the dissection of autoimmune diseases, optimization of cancer treatment, and guidance of vaccine development [110].

### Performance characteristics and optimization strategies of RNA sequencing

#### Length

By “length,” we refer to the length of the raw reads output by the sequencer. In contrast to short reads of NGS, LRS platforms produce substantially longer reads, with continuous improvements being made over the years. For transcriptomics, both PacBio and ONT can capture full‐length transcripts for the vast majority of RNA biotypes, as most transcripts are less than 10 kb (typically 1–2 kb in human and mouse) [40], while the performance of other LRS platforms remains to be assessed. Most studies focus on the sequencing lengths achieved by LRS platforms in the context of genome sequencing. PacBio platforms typically generate reads of 10–30 kb, with consensus sequence length strongly dependent on library insert size and the sequencing strategies employed (CLR or HiFi modes) [49]. For ONT‐based genome sequencing, read length is largely determined by the length and integrity of the extracted genomic DNA. The typical read lengths range from 5 to 50 kb [111], while the longest reported reads reached 2.3 Mb in a third‐party study [112] and 4.2 Mb in‐house at ONT. Despite the multi‐kilobase capabilities of PacBio and ONT, cDNA‐based library preparation biases (e.g., RNA fragmentation during RT, size selection, preferential amplification of shorter molecules) systematically underrepresent very long transcripts (e.g., >10 kb). On the other hand, the practical performance of DRS is constrained by RNA structure and motor protein dynamics [113, 114]. The median length of DRS reads typically ranges from 0.8 to 1.3 kb [113, 115]. A key limitation is systematic 5' end signal loss (~10–15 nt) caused by premature motor protein dissociation [113].

Although other LRS platforms claim the ability to generate long reads (e.g., >100 kb), independent third‐party evaluations are lacking. As reported in 2022, QitanTech's QNome platform achieved read lengths of up to ~150 kb [30]. CycloneSEQ has reported a mean read length of 19.2 kb in human whole‐genome sequencing [31]. In the future, it will be necessary to assess whether these platforms can reliably capture the 5' and 3' ends of transcripts, as many sequencing techniques suffer from signal noise that obscures terminal regions. Benchmarking studies assessing end‐to‐end coverage across full‐length transcripts are therefore essential.

#### Accuracy

By “accuracy,” we refer to the accuracy of base calling. LRS platforms suffer higher error rates compared with NGS [116]. In genomic DNA sequencing, PacBio's CLR mode can generate long reads but has a high error rate of 13–15% [117], whereas its HiFi mode achieves 99.9% consensus sequence accuracy [18, 28]. Our previous benchmark study demonstrated 98% accuracy for Iso‐Seq data [118]. Historically, early ONT runs exhibited high error rates: 40% for genomic DNA sequencing [111] and 20% for cDNA sequencing [118]. To address this, ONT continuously updated its sequencing chemistries and basecalling algorithms [119]. The R10.4 chemistry achieved a modal accuracy of >99% for genomic DNA sequencing [120]. Despite these substantial improvements, ONT generally still trails PacBio HiFi in single‐read accuracy. Mismatches remain the predominant error type in ONT data, featuring strong context specificity, while insertion and deletion errors are heavily biased towards homopolymeric regions [118].

To improve accuracy for cDNA sequencing, the Rolling Circle to Concatemeric Consensus (R2C2) method was developed to leverage the long sequencing lengths of the ONT platform for generating consensus sequences with higher accuracy (Figure 2E). R2C2 introduces pre‐sequencing redundancy by circularizing full‐length cDNAs and amplifying them via Phi29‐mediated rolling circle amplification (RCA) into long concatemers with multiple tandem repeats of the target insert [121, 122]. It combines RCA with concatemeric consensus calling: parsing and collapsing these repeat units into high‐accuracy consensus sequences post‐sequencing. This approach reduces random sequencing errors and minimizes interference from truncated molecules in the final dataset [40]. R2C2 improves median read accuracy from 87% to 94% for ONT cDNA sequencing [122]. This accuracy gain comes with a trade‐off of reduced effective sequencing depth and throughput, as sequencing capacity is used to generate the redundant repeats required for consensus calling. Nevertheless, R2C2 features high multiplexing potential, cost‐effectiveness, and compatibility with established cDNA workflows, supporting its use in both bulk and single‐cell full‐length transcriptome analyses [40, 121].

In terms of ONT DRS data accuracy, early chemistries exhibit accuracies below 90% [123, 124]. The RNA004 chemistry improves the median read accuracy to 93.5% [125], and its combination with the Dorado basecalling algorithm achieves a median accuracy of 98.67% [126].

#### Throughput

By “throughput,” we refer to the number of sequenced reads produced per experimental run. To accommodate diverse research demands and reduce sequencing cost, LRS companies have released different devices/platforms with different throughput capacities (Figure 1A).

ONT offers a range of sequencers: MinION (low throughput), GridION (medium), and PromethION (high) [13]. MinION is a portable device that can run a MinION flow cell featuring 512 channels, each with one active nanopore, enabling concurrent sequencing of up to 512 molecules [127, 128]. GridION is a medium‐scale device capable of running five MinION flow cells simultaneously. PromethION accommodates 24 or 48 parallel PromethION flow cells, each containing 2675 channels, with the yield per PromethION flow cell approximately five times that of a MinION flow cell [50]. A single PromethION flow cell typically produces 50–150 gigabases (Gb) of data for PCR‐cDNA sequencing (50–150 million reads with an average length of ~1 kb per read) and 10–30 Gb of data for DRS (10–30 million reads with an average length of ~1 kb per read) [38, 129]. The Flongle flow cell with 126 channels, designed for smaller‐scale applications, has been discontinued. For researchers seeking small‐scale or targeted applications, adaptive sampling/sequencing can serve as an alternative [130, 131]. Future strategies to increase ONT throughput may involve new motor proteins with higher translocation speeds, though this could potentially compromise accuracy.

PacBio has progressively upgraded its devices to increase throughput, evolving from the initial RS (150,000 ZMWs) and RS II (150,000 ZMWs) to the Sequel (1 million ZMWs), Sequel II (8 million ZMWs), and the latest high‐throughput Vega (25 million ZMWs) and Revio (25 million ZMWs) [132]. Revio system can run four SMRT Cells, totaling 100 million ZMWs [133]. Since each ZMW can be used to sequence one molecule per run, read count serves as a more informative metric for PacBio throughput. Because the SMRT Cell cannot be overloaded, the proportion of ZMWs that successfully undergo sequencing is about 40%–60% [46]. A typical Revio or Vega SMRT Cell yields 6–8 million HiFi reads [134]. In the traditional PacBio Iso‐Seq workflow, cDNA templates are far shorter than the platform's maximum read length, leading to substantial waste of capacity utilization, as each ZMW sequences only one molecule [135, 136]. Therefore, developing library preparation strategies that boost throughput while preserving the long‐read advantages of PacBio has become critical for promoting its large‐scale applications.

Kinnex, a recent advancement in the PacBio sequencing ecosystem, is the commercial implementation of Multiplex Any Sequence (MAS‐Seq, termed MAS‐ISO‐seq when applied to transcriptome sequencing) principles for substantial throughput enhancement [134, 137] (Figure 2F). The core mechanism of Kinnex/MAS‐ISO‐seq involves concatenating multiple short cDNA fragments (eight fragments in the current version of Kinnex) into segmented long templates (15–20 kb) using dedicated adapters, adapted from the deoxy‐uracil (dU) barcode adapter technology of MAS‐ISO‐seq [134]. Following sequencing, the long templates are bioinformatically demultiplexed to recover the sequence information of individual short cDNA templates without compromising integrity or accuracy. Kinnex maintains PacBio's hallmark HiFi accuracy (99.9%) while delivering an eightfold throughput increase for transcriptome sequencing [137, 138]. Leveraging the same cDNA concatenation strategy, HIT‐scISOseq is a specialized high‐throughput single‐cell transcriptome sequencing method developed for PacBio. A single Sequel II SMRT Cell 8M using HIT‐scISOseq can generate more than 10 million high‐accuracy reads [139].

Other LRS companies have also released devices with varying throughputs. QitanTech offers QNome (low), QPursue (medium), and QPinnacle (high). CycloneSEQ has released G100‐ER (medium) and G400‐ER (high). Polyseq provides PolyseqOne (low), PolyseqHive (medium), and PolyseqX2 (high). Geneus offers G‐seq. 500 (low) and G‐seq. 1 M (high). Although these companies claim that their platforms can generate various throughputs, the quantity and quality of sequencing data remain to be evaluated by independent third‐party studies in the future. While platform throughput defines the upper bound of sequencing output, the required sequencing depth for long‐read transcriptomics is highly dependent on the biological question and transcriptome complexity. In human samples, approximately 10 million long reads are considered sufficient to recover a large fraction (80%) of known isoforms [140], and 20 million long reads are suggested for accurate transcript quantification [141]. Subsampling‐based saturation analyses are recommended to define the optimal data size for different organisms. Data size guidelines for single‐cell and spatial long‐read transcriptomics remain under active development, as they depend on factors such as cell or spot number, capture efficiency, barcode recovery, and reads per cell or spot.

## BIOINFORMATICS METHODS: FROM RAW SIGNALS TO TRANSCRIPTS

### Data preprocessing: Basecalling, quality control, and error correction

Primary data processing mainly encompasses basecalling, quality control (QC), and error correction, serving as a critical intermediate step that bridges raw sequencing data (Figure 3A) and downstream analyses (Table S3).

**FIGURE 3:**
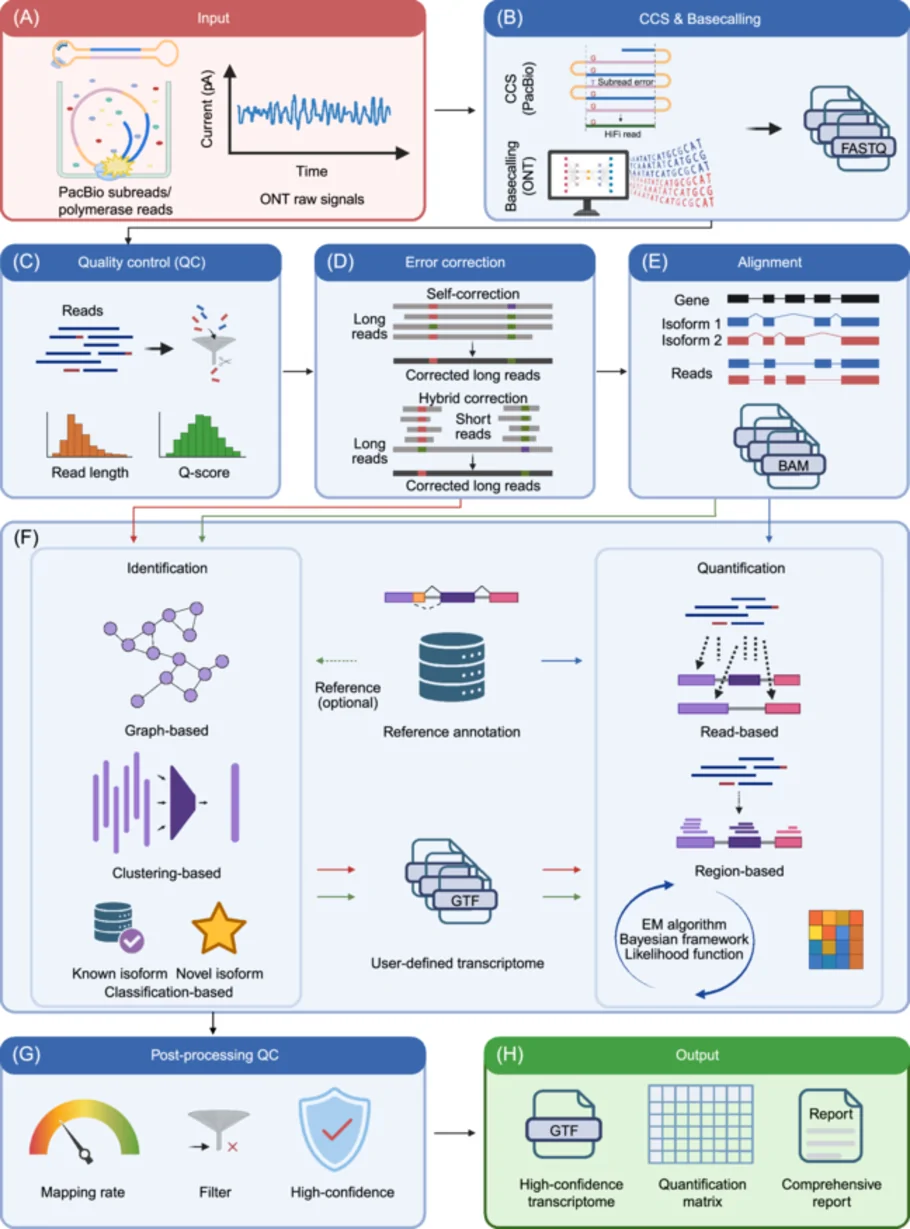
Computational workflow for long‐read transcriptome data analysis. (A) PacBio subreads/polymerase reads and ONT raw current signals are input data for platform‐specific preprocessing. (B) PacBio subreads/polymerase reads are processed to generate CCS, which combines repeated observations of the same molecule to reduce subread errors and generate high‐accuracy HiFi reads. ONT raw signals are converted into nucleotide sequences through basecalling. The resulting PacBio and ONT reads are then converted to FASTQ format for QC. (C) QC filters reads according to quality‐related metrics, such as read length and Q‐scores, to remove low‐quality sequences/bases before downstream analyses. (D) Error correction improves long‐read accuracy through self‐correction using overlapping reads or hybrid correction integrating short‐read data. (E) Alignment maps high‐quality reads to a reference, typically a reference genome, to produce alignment files (BAM) and to resolve exon‐intron structures and isoform‐specific read mappings. (F) Transcript identification and quantification can be executed through three distinct strategies, indicated by colored arrows. The red pathway represents a reference‐free inference strategy, which directly employs quality‐controlled reads for *de novo* transcript reconstruction and quantification without requiring a reference genome alignment, making it particularly suitable for species lacking reference genomes. The green pathway illustrates a reference‐based inference approach, where aligned reads are utilized to infer transcript models via graph‐based, clustering‐based, or classification‐based methods. This strategy distinguishes between known and novel isoforms, generating a user‐defined transcriptome (GTF) for subsequent quantification. The blue pathway depicts an annotation‐driven method, which bypasses the novel transcript identification step and directly quantifies expression based on existing reference annotations; this provides a more computationally efficient route but precludes the discovery of novel isoforms. During the identification process, reference annotation can optionally be used to guide transcript model construction. Quantification relies on read‐based or region‐based strategies and commonly uses probabilistic frameworks, such as EM algorithms, Bayesian models, or likelihood‐based inference, to generate expression estimates. (G) Post‐QC evaluates mapping and quantification results using metrics such as mapping rate, mapping quality thresholds (MAPQ), and confidence filters to retain high‐confidence transcript models and expression estimates. (H) The final outputs include a high‐confidence transcriptome annotation (GTF), a transcript/isoform quantification matrix, and a comprehensive analysis report. CCS, circular consensus sequencing; QC, quality control; EM, expectation‐maximization; pA, picoampere; Q score, Phred quality score. Created in BioRender. Li, J. (2026) https://BioRender.com/no4nlqs.

### Basecalling

The basecalling of PacBio and ONT starts from fundamentally different signal modalities, which drives different algorithmic priorities (Figure 3B). ONT converts raw ionic‐current time series generated as RNA or DNA molecules translocate through a nanopore into nucleotide sequences. Because each current state reflects a short local sequence context rather than a single nucleotide, basecalling is best framed as decoding a noisy continuous signal into a discrete sequence [13]. Accordingly, ONT has evolved from event segmentation plus Hidden Markov Model (HMM)‐based decoding to end‐to‐end deep learning directly on raw signals, including Convolutional Neural Networks (CNN) [142], Recurrent Neural Networks (RNN) [143], attention‐based networks [144], and Transformer models [145], typically combined with Connectionist Temporal Classification‐style decoding [146]. Importantly, the same signal‐generation principle also underlies ONT‐based modification detection during or immediately after basecalling. Chemically‐modified DNA/RNA (e.g., DNA 5mC, RNA m^6^A) bases can perturb the expected ionic‐current pattern of the corresponding canonical bases. In ONT DRS, native RNA molecules pass through the pore, allowing modified bases (e.g., m^6^A, m^5^C, Ψ, inosine) to generate deviations in current intensity, dwell time, signal variance, basecalling quality, or error profiles [147, 148, 149]. These deviations can be interpreted as modification signatures by comparing observed signals with expected canonical k‐mer models, testing differential signal distributions between modified and unmodified or perturbed samples, or training machine‐learning models on signal‐derived features from known modified sites [150, 151, 152, 153].

PacBio, by contrast, records optical pulses and polymerase‐kinetic signals during synthesis. Here, basecalling is closely coupled to CCS: multiple subreads from the same molecule are aggregated into a high‐accuracy HiFi read [154]. As a result, ONT accuracy is largely driven by better single‐pass signal decoding, whereas PacBio accuracy is primarily determined by multi‐pass consensus generation, increasingly improved by deep learning‐based consensus refinement (e.g., DeepConsensus [155]).

Algorithmically, ONT basecalling can be organized into three practical classes. First, event‐level HMM approaches [156, 157] are relatively interpretable but can be limited by information loss introduced during segmentation. Second, raw‐signal deep learning methods (CNN [142]/RNN [143]/temporal convolutional network [158]/Transformer [145]/Conformer families [146]) substantially improve accuracy by capturing both local patterns and longer‐range dependencies, but they require more computational resources and careful matching to pore chemistry, library type, and training domain. Third, modification‐aware frameworks [159], as noted above, infer RNA modifications from signal features during or immediately after basecalling; this is a major advantage for DRS, but performance can be sensitive to training data, chemistry, and sequence context. In the current ONT ecosystem, Dorado (https://github.com/nanoporetech/dorado) is a widely used and optimized baseline, while third‐party tools such as RODAN [160], Coral [161], SqueezeCall [146], BaseNet [145], DemuxTrans [162], DEMINERS [163], and GCRTcall [164] explore alternative architectures and may outperform on specific datasets (several are designed for DRS). For PacBio, the most useful distinction is between standard signal decoding followed by CCS/HiFi consensus generation and deep learning‐enhanced consensus refinement (notably DeepConsensus [155]). This consensus‐centric strategy yields very high final read accuracy, which is particularly beneficial for splice‐junction precision, isoform reconstruction, and transcript annotation, but it is generally less direct than ONT for native RNA modification analysis.

Practically, Dorado (https://github.com/nanoporetech/dorado) is a reasonable starting point for ONT RNA‐seq workflows because of its official ONT support, chemistry‐specific models, and hardware‐accelerated implementation, with RNA modified‐base calling available for supported DRS models. Alternative basecallers should be evaluated using representative data matched to the sequencing chemistry, sample composition, and downstream task, with selection guided by accuracy, computational cost, and required functionality. PacBio RNA‐seq workflows should prioritize the latest CCS/HiFi pipeline and incorporate DeepConsensus [155] when available, because consensus quality is the main determinant of downstream performance. Overall, PacBio is typically preferred when maximum sequence accuracy and isoform resolution are the primary goals, whereas ONT is often preferred for native RNA‐seq and modification‐aware applications.

### Quality control

Although recent technological advances have markedly reduced per‐read error rates, long‐read platforms still exhibit platform‐specific biases and technical variability. Therefore, rigorous QC workflows and effective data visualization, spanning from raw data assessment to transcriptome assembly, remain essential to ensure reliable downstream analyses (Figure 3C).

Raw read QC is the first step of long‐read transcriptome analysis. Common cross‐platform metrics include total yield, read length distribution, base quality scores, GC content, and adapter/primer contamination [165]. Transcriptome‐oriented libraries benefit from additional checks for full‐length enrichment and chimeric artifacts. Platform‐specific indicators further improve interpretability [121]. In PacBio Iso‐Seq, the fraction of full‐length non‐chimeric (FLNC) reads is a key measure of library quality and size‐selection performance [166]. In ONT, time‐resolved yield and sequencing‐summary statistics are useful for diagnosing loading efficiency, pore performance, and run stability [167].

To interpret QC metrics effectively, it is essential to understand the failure modes that each indicator reveals. For long‐read transcriptome data, common issues manifest as distinct signatures. A sharp drop in read length compared to expected insert size often points to shearing during library preparation or suboptimal size selection. An excess of very short reads (<500 bp) alongside low FLNC fractions suggests RNA degradation or inefficient RT.

QC tools can be selected based on function and platform. For consistency, reference‐free summaries across ONT and PacBio, LongQC [165] and LongReadSum [168] provide core statistics on yield, read length, and quality distributions. For visualization, NanoPack2 (including NanoPlot and related modules) [169] generates standard plots such as read‐length histograms, length‐quality scatter plots, GC‐content distributions, and yield‐over‐time profiles, which makes it easier to spot outliers and compare runs. For ONT‐specific monitoring and reporting, MinKNOW [170] supports real‐time run tracking and control, while pycoQC [171] converts sequencing summary files into interactive HTML reports for post‐run review. For preprocessing that combines QC with cleaning, fastplong [172] (https://github.com/OpenGene/fastplong) integrates QC reporting with read filtering, adapter trimming, and poly(A)‐tail processing, which is well suited to full‐length cDNA and transcriptome‐focused workflows.

In practice, a recommended practice is to apply a cross‐platform summary tool (LongQC [165] or LongReadSum [168]) and a visualization tool (NanoPlot [169]) to every dataset to establish a comparable QC baseline. For ONT data, include MinKNOW [170] during sequencing and pycoQC [171] after sequencing to evaluate run health and diagnose common failure modes; use fastplong [172] (https://github.com/OpenGene/fastplong) when trimming, filtering, or poly(A) processing is required before mapping or assembly. For PacBio Iso‐Seq, prioritize FLNC‐related checks from the standard Iso‐Seq processing outputs, and complement them with LongQC/LongReadSum plus NanoPlot to confirm that yield, length, and quality distributions match expectations. This combination ensures that QC remains both platform‐aware and end‐to‐end, reducing the risk that early technical issues compromise transcriptome reconstruction and downstream interpretation.

### Error correction

LRS provides long read length, but suffers from sequencing errors, often enriched for insertions and deletions [173]. Error profiles differ across platforms (more random in PacBio, more sequence‐ and context‐biased in ONT), and low‐accuracy read subsets can persist even when average accuracy improves [174]. Without correction, these errors reduce mappability, blur splice junctions and breakpoints, inflate spurious isoforms, and increase misassemblies, ultimately compromising downstream inference [175]. Signal‐level correction is addressed in the basecalling section. Here we focus on correction methods that operate solely on read sequence data (Figure 3D).

Methodologically, correction tools can be grouped by the input data they rely on and the models used to derive a consensus. Hybrid correction uses accurate short reads and typically achieves the lowest residual error; alignment‐based methods (e.g., PacBioToCA (https://rhallpb.github.io/Applications/pacBioToCA.html), proovread [176], and Hercules [177]) are accurate but can be computationally heavy and sensitive to ambiguous mappings, whereas de Bruijn graph‐based methods (e.g., LoRDEC [178], Jabba [179], FMLRC [175], and HALC [180]) are often more scalable but depend on sufficient short‐read depth and can be challenged by repeats and complex variation. Self‐correction relies only on long reads, using overlap‐consensus approaches (e.g., Canu [181], MECAT [182]/NECAT [183], CONSENT [184], and NextDenovo [185]) or graph‐based strategies (e.g., LoRMA [186]); it enables long‐read‐only workflows but usually leaves higher residual error, particularly when systematic ONT errors dominate.

For UMI‐tagged single‐cell and spatial protocols, error correction can additionally leverage molecule‐level redundancy, but the effectiveness depends largely on how UMIs are recovered and utilized [187]. In principle, collapsing reads originating from the same UMI reduces both sequencing and PCR errors [188]; however, UMI recovery itself is non‐trivial in long‐read data due to sequencing errors that can fragment true UMI groups [189]. Recent methods have been developed to address this challenge, see later discussion under “Frontiers: Long‐read single‐cell transcriptomics”. With the recovered UMI, mapping and truncation errors in the sequencing reads can be corrected by assigning reads to UMI groups and applying majority voting within each UMI group [190].

In practice, the choice of long‐read error correction should be guided by data availability, platform error characteristics, and downstream objectives. When matched high‐accuracy short reads are available, hybrid correction is generally preferred for both ONT and PacBio; FMLRC [175] and LoRDEC [178] offer time‐ and memory‐efficient solutions for processing large‐scale human sequencing datasets [191]. When only long reads are available, self‐correction is recommended and benefits from sufficient coverage; Canu [181] and NECAT [183] are common defaults for assembly‐oriented genomic datasets, with CONSENT [184] as an additional consensus‐based option. For UMI‐tagged single‐cell/spatial long‐read transcriptomics, molecule‐level UMI‐aware consensus correction should be prioritized, with FLAMES [121] recommended. For ONT datasets with pronounced context‐specific errors or heterogeneous read quality, deep learning tools such as DeepCorr [192] and NmTHC [193] may be evaluated as supplementary approaches, ideally validated on the same chemistry and basecalling configuration. In addition to read‐level correction, modern long‐read workflows often incorporate reference‐guided polishing or variant‐aware refinement steps. For ONT data, Medaka (https://github.com/nanoporetech/medaka) is widely used for neural‐network‐based consensus polishing after read mapping, helping to reduce residual systematic errors, particularly indels in difficult sequence contexts. Small‐variant callers such as Clair3 [194] and DeepVariant [195] are not read‐correction tools in the strict sense, but they are increasingly used in long‐read analysis pipelines to identify single nucleotide polymorphisms (SNPs) and indel errors, benchmark sequence accuracy, and support high‐confidence consensus refinement when a reference genome is available. Clair3 combines pileup and full‐alignment information in a deep‐learning framework optimized for long reads, whereas DeepVariant uses deep neural networks to call SNPs and small indels from aligned read evidence across multiple sequencing platforms. For transcriptomic applications, Clair3‐RNA [196] further extends this framework to LRS by introducing improvements tailored to transcriptomic data, including better handling of splice‐aware alignments and RNA‐specific sequence contexts, thereby improving variant calling accuracy in expressed regions. These tools therefore complement sequence‐based correction methods, especially in reference‐guided analyses where accurate small‐variant resolution is important. In independent benchmarks, FMLRC, Canu, and FLAMES perform consistently well [173, 191, 197]. However, for allele‐specific analyses, error correction should be applied cautiously because it can eliminate allelic information by correcting heterozygous sites [198].

### Identification and quantification of gene transcripts

Transcript identification and quantification are core tasks in transcriptomic analyses (Figure 3E,F). In reference‐guided workflows, reads are mapped to a reference genome and/or transcriptome, and transcript models are inferred by assigning reads to annotated isoforms or by reconstructing novel isoforms from the alignments. When an appropriate reference genome is unavailable, de novo approaches assemble transcripts directly from read‐to‐read sequence relationships rather than from genomic coordinates. For quantification, abundance estimates are computed with respect to a defined set of features (e.g., annotated transcripts, reconstructed isoforms, genes, or exons); thus, quantification depends on the feature definition, even when a tool does not explicitly report a separate identification output. Although many current pipelines integrate read alignment (or pseudoalignment), transcript identification, and transcript quantification, these components are described separately below for clarity, while noting that choices in earlier steps constrain and propagate to downstream estimates (Figure 3).

### Read alignment

Aligning long reads to a reference genome is challenging because reads are long and error‐prone, and often span multiple exons. The aligners should be both splice‐aware and tolerant of indels while still placing exon boundaries precisely [199] (Figure 3E). Key failure modes include ambiguous placement in repetitive or paralogous regions, misassignment of short or micro‐exons, and false or shifted splice junctions, issues that are exacerbated by platform‐specific error patterns (e.g., ONT indel bias) and by complex isoform diversity and low‐abundance transcripts [200]. Practical considerations, therefore, include using splice‐aware settings, selecting presets matched to library type (cDNA vs. direct RNA) and platform, and visually inspecting borderline loci in genome browsers when transcript models depend on junction‐level accuracy [201].

Most current long‐read aligners follow a seed‐chain‐extend paradigm [201], differing mainly in their indexing and how they handle errors and splicing. Hashing/minimizer‐based methods dominate in practice: minimap2 is the de facto standard due to its speed, versatility, and strong splice‐aware alignment performance, with presets that simplify optimization (e.g., ‐ax splice:hq for high‐quality PacBio Iso‐Seq, ‐ax splice for ONT cDNA, and ‐ax splice ‐uf ‐k14 for ONT direct RNA) [202]. However, minimap2 can still struggle with small exons, boundary precision in noisy reads, and spurious splice sites in difficult sequence contexts. Several tools target these limitations: Graphmap2 improves recall and can place exon/transcript boundaries more precisely [203], while deSALT uses a de Bruijn graph‐based index to better tolerate sequencing errors and recover small exons [204]. BWT‐FM‐index approaches enable efficient exact‐match retrieval with inexact extension; BWA‐MEM2 is a fast general‐purpose aligner (not primarily splice‐centric) that supports local/end‐to‐end and chimeric alignments [205], and uLTRA emphasizes loss‐free MEM retrieval that can help with small exons and complex splice sites [206]. Suffix‐array approaches (e.g., STAR [207]) are less commonly used for long reads because uncompressed indices are memory‐heavy and long‐read error profiles reduce seeding efficiency, although the core strategy can be extended to long‐read mapping [208, 209].

For most long‐read transcriptome studies, a practical choice is minimap2 [202] in splice‐aware mode with the correct preset for the platform/library, followed by splice‐junction refinement when junction accuracy is critical. For example, 2passtools [210] uses a two‐pass strategy with machine‐learning‐filtered junctions to improve intron detection, whereas Splam [211] applies a deep‐learning‐based splice‐site model to score junctions from spliced alignments and flag low‐confidence or spurious events, thereby reducing false splice junctions introduced by alignment errors, particularly in noisy ONT reads. Such post‐alignment correction/filtering can be especially valuable for high‐confidence novel isoform discovery, where even small junction shifts may inflate artifactual transcript models. FLAIR‐correct [212] provides another widely used option to correct splice sites from minimap2 alignments using reference annotation and short‐read evidence when available. Consider deSALT [204] when small exons and high error rates are prominent, and Graphmap2 [203] when boundary precision/recall is prioritized over speed. Further improvements are likely to come from better seeding and chaining in repetitive regions and regions rich in structural variation (SV), explicit support for haplotype‐aware and pangenome/graph references, and tighter integration of alignment with learned error models and post‐alignment junction validation to reduce systematic splice artifacts.

### Transcript identification

Long‐read transcriptomics has made transcript identification a central task because full‐length (or near full‐length) reads can directly resolve exon connectivity and enable discovery of novel isoforms, which are absent from existing annotation libraries such as GENCODE [199] (Figure 3F). The main challenges are also long‐read‐specific: sequencing and alignment noise around splice junctions, variable read truncation and end biases (i.e., uncertainty in transcription start site (TSS) and transcription termination site (TTS)), artifacts during library preparation (e.g., RT switching and intra‐priming) that generate false‐positive novel isoform calls, and the fact that true novel isoforms are often lowly expressed and difficult to distinguish from errors (Table S3). Conceptually, current methods fall into two broad strategies: reference genome‐guided identification (reference genome available; optionally using existing gene annotations) and reference genome‐free/*de novo* identification (no reliable reference genome) [18]. Across both strategies, most tools can be grouped by core algorithmic ideas into graph‐based, clustering‐based, and class/annotation‐based approaches, often embedded in end‐to‐end workflows that combine identification, annotation, and quality control (Figure 4).

**FIGURE 4:**
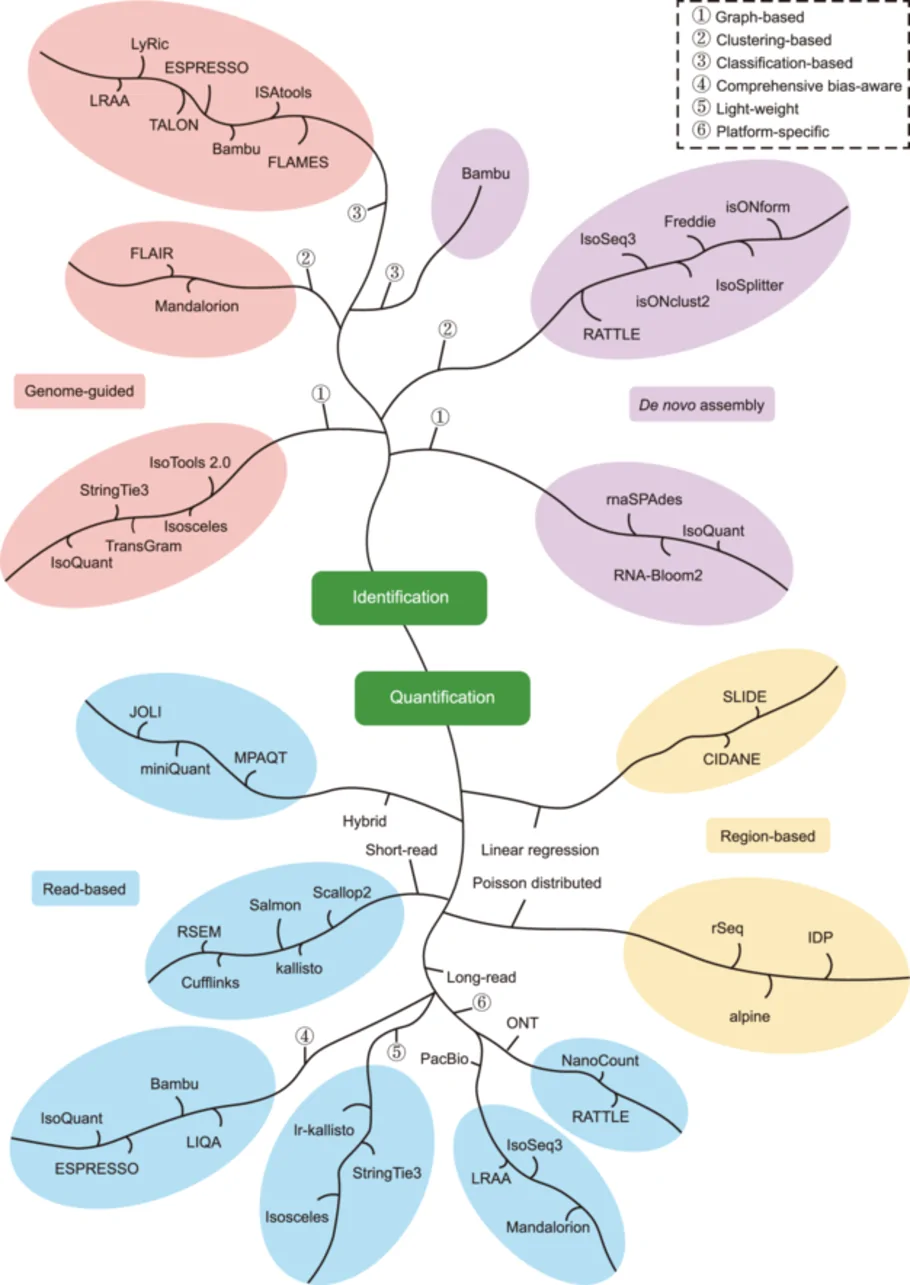
Methodological phylogeny of computational tools for transcript identification and quantification. This diagram organizes representative software for LRS‐based transcript identification and quantification according to methodological similarity. The upper part summarizes isoform identification strategies, including genome‐guided and de novo assembly approaches. Identification methods are further grouped by core algorithmic paradigms: (1) graph‐based, (2) clustering‐based, and (3) classification‐based frameworks (numbers on branches). The lower part summarizes isoform/transcript quantification methods, separating read‐based and region‐based models and highlighting different data regimes (short‐read, hybrid, and long‐read). Long‐read quantification methods are further classified as (4) comprehensive bias‐aware, (5) lightweight, and (6) platform‐specific approaches. Labels such as Poisson‐distributed and linear regression denote typical statistical assumptions used by subsets of tools. Tool names are placed within each module to illustrate representative implementations under each methodological class. ONT, Oxford Nanopore Technologies; PacBio, Pacific Biosciences.

With a reference genome (the dominant setting for human and mouse), methods start from splice‐aware genome alignments and then reconstruct isoforms using (i) graph‐based models (e.g., StringTie3 [213], IsoQuant [214], IsoTools 2.0 [215], Isosceles [216], Transgram (https://github.com/yutingsdu/TransGram)) that infer transcript paths from splice graphs; these can be accurate and expressive for complex splicing but may over‐generate isoforms without strong constraints and filtering. (ii) Clustering‐based approaches (e.g., FLAIR [212], Mandalorion [217]) group reads by shared splice‐junction chains and derive isoform models from read clusters; they can be robust to sparse isoforms but depend heavily on alignment quality and junction correction. (iii) Class/annotation‐based approaches (e.g., ESPRESSO [218], TALON [219], FLAMES [121], Bambu [220], LRAA [221], ISAtools [222]) classify models into known/novel categories, namely Full Splice Match (FSM), Incomplete Splice Match (ISM), Novel In Catalog (NIC), and Novel Not in Catalog (NNC) [223] and typically emphasize splice correction and structured filtering; they offer strong interpretability and better false‐positive control, but conservative settings can reduce sensitivity for rare/low‐expression novel isoforms. Overall, reference‐guided methods are computationally efficient and best suited to well‐annotated organisms, yet still require careful end/junction validation to avoid spurious novelty.

When no reliable reference genome and/or annotation is available, transcript identification shifts to de novo reconstruction from read overlap, sequence similarity, or assembly graphs. Graph/assembly‐based methods (RNA‐Bloom2 [224], rnaSPAdes [225]) can maximize discovery and often improve transcriptome completeness in non‐model settings, but tend to trade precision for sensitivity, with higher fragmentation and more non‐canonical junctions requiring stringent downstream curation. Clustering/consensus‐based methods (IsoSeq. 3 (https://github.com/ylipacbio/IsoSeq. 3), isONclust2 (https://github.com/nanoporetech/isonclust2), RATTLE [226], isONform [227], Freddie [228], IsoSplitter [229]) attempt to reconstruct isoforms by grouping similar reads and refining consensus sequences; they can yield high‐confidence models when read accuracy is high (notably PacBio CCS/HiFi in IsoSeq. 3‐style workflows), but may underperform on noisier data or highly complex loci unless supported by strong error correction and validation. Finally, workflow‐style solutions such as LyRic (https://github.com/guigolab/LyRic) focus on producing a curated set of genome‐mapped transcript models plus reports/track hubs; they are practical for reproducible annotation building, but their novelty/precision balance is driven by internal filters and any orthogonal evidence that is enabled.

Recent benchmarking provides actionable guidance on method choice by data regime. In bulk ONT mixtures with spike‐ins, StringTie3 [213] and Bambu [220] were top performers for isoform detection overall, while the same study highlighted persistent overcalling of novel junctions and the value of short‐read junction support for filtering [41]. The community LRGASP assessment concluded that for well‐annotated genomes, reference‐based tools are generally best; when minimal novelty is expected, Bambu [220], IsoQuant [214], and FLAIR [212] are effective choices, whereas detecting rare/lowly expressed transcripts benefits from tools that allow novelty and incorporate orthogonal data and replicates. In reference‐free de novo settings, RNA‐Bloom2 [224] tended to be more sensitive (useful for exploratory catalogs), Bambu [220] more conservative (high‐confidence cores), and rnaSPAdes [225] produced many fragmented models and exhibited a high false discovery rate (FDR), implying it should be paired with strict filtering [40]. For single‐cell/spatial ONT, benchmarking showed that upstream barcode/UMI correction strongly conditions isoform discovery and quantification; wf‐single‐cell (https://github.com/epi2me-labs/wf-single-cell) provided HiFi isoform quantification in simulations, while Bambu [220] and Isosceles [216] were strong for novel isoform discovery under specific error/chemistry regimes, and IsoQuant [214] was notably more scalable than Isosceles [216] in runtime and memory [44]. Finally, the community SG‐NEx demonstrated experimentally that fragmentation can inflate short‐read major isoform calls, reinforcing the practical recommendation that when the biological question hinges on dominant isoforms or long‐range coupling of promoter‐splicing‐poly(A), long‐read‐based identification (with junction/end QC) should be prioritized [42].

### Transcript quantification

Transcript quantification estimates isoform abundances from sequencing reads (Figure 3F). Its fundamental challenge is identifiability: because isoforms often share exons and splice junctions, many reads are compatible with multiple transcripts. This ambiguity is further compounded by fragmentation, 3′/5′ end bias, truncation, PCR effects, and sequencing or alignment errors [18, 230, 231]. To address these issues, most methods use probabilistic generative models and infer abundances by maximum likelihood (*e.g*., kallisto [232], RSEM [233], Salmon [234], LIQA [235], and miniQuant [141]), typically with Expectation‐Maximization (EM) to distribute ambiguous reads across compatible isoforms, whereas region‐based methods (*e.g*., rSeq [11], IDP [236], and alpine [237]) use generalized linear model frameworks under stronger simplifying assumptions (Figure 4). In practice, short reads provide high depth and low error rates but suffer from substantial isoform ambiguity, whereas long reads improve identifiability by spanning multiple splice junctions and directly supporting transcript structures. However, long reads also introduce protocol‐specific biases, particularly 3'/5' bias and truncation, and ONT data further present higher error rates that can impair splice alignment. Recent benchmarks consistently highlight this trade‐off: read length and accuracy mainly improve transcript identification, whereas sequencing depth mainly improves quantification accuracy [40].

In this context, long‐read quantification methods can be broadly categorized by how they model long‐read‐specific artifacts and how they assign reads to isoforms. Bias‐aware probabilistic methods (e.g., IsoQuant [214], Bambu [220], ESPRESSO [218], and LIQA [235]) explicitly model factors such as sequencing error, effective length, truncation, and end bias, and usually rely on EM soft assignment. These methods are generally more robust for complex loci and ambiguous read compatibility, although they depend more heavily on accurate alignments and annotations. Platform‐optimized pipelines (e.g., IsoSeq. 3 (https://github.com/ylipacbio/IsoSeq. 3), Mandalorion [217], NanoCount [115], RATTLE [226], JOLI [238], and LRAA [221]) often incorporate upstream correction or consensus generation and then apply simpler counting or hard assignment strategies. These approaches are efficient and interpretable when isoforms are well separated, but they are more prone to read misallocation when transcripts are highly similar or coverage is limited. Lightweight methods (*e.g*., Isosceles [216], lr‐kallisto [239], and StringTie3 [213] in quantification mode) simplify compatibility representations and/or correct only dominant biases to scale efficiently to large datasets, but they may be less stable for genes with high isoform ambiguity unless transcript definitions are reliable and quality control is stringent. By contrast, assembly‐first workflows (e.g., TALON [219], FLAMES [121], FLAIR [212], IsONform [227], IsoSplitter [229], Oarfish [240], and ISAtools [222]) prioritize transcript‐model construction and filtering before quantification. While attractive for transcript discovery, their quantification accuracy is constrained by reconstruction quality, so spurious novel isoforms can directly inflate abundance estimates.

These limitations have motivated the integration of long‐ and short‐read sequencing. In such hybrid strategies, long reads reduce structural ambiguity and help define the expressed isoform repertoire, whereas short reads contribute the depth needed for more precise abundance estimates. This combination is particularly valuable when short‐read fragmentation distorts apparent transcript usage or when long‐read coverage is insufficient to stabilize estimates for low‐abundance isoforms. Reflecting this trend, recent methods increasingly combine short‐read depth with long‐read structural constraints in unified frameworks, as exemplified by miniQuant [141], or borrow information across samples to improve estimation stability, as in empirical Bayesian approaches such as JOLI [238] and MPAQT [241].

As long‐read and hybrid analyses become more common, quantification uncertainty should be treated as a central result rather than a secondary issue. Ambiguous read assignment, low expression, end bias, truncation, and alignment errors all increase variance and can propagate into downstream analyses such as differential transcript usage. To quantify gene‐specific stability, miniQuant [141] introduces a *K*‐value (*e.g*., the generalized condition number) defined from the read‐isoform compatibility matrix *A* as the ratio of its largest to smallest positive singular value. Larger *K*‐value indicates poorer conditioning and therefore greater potential relative quantification error under a given read‐length regime. Based on this metric, miniQuant [141] further uses machine learning‐based strategies to adaptively weight long‐ and short‐read evidence. The *K*‐value thus provides a practical indicator of when abundance estimates are intrinsically unstable and when additional sequencing depth or alternative experimental designs may be necessary.

Consistent with these observations, benchmark‐driven recommendations are increasingly scenario‐specific. For bulk RNA‐seq in well‐annotated genomes, LRGASP [40] shows that performance depends strongly on sequencing protocol and depth: among long‐read tools, IsoQuant [214], FLAIR [212], and Bambu [220] consistently perform well, whereas short‐read methods may still outperform when annotation quality and sequencing depth are both favorable. For transcript identification, which sets the upper limit for quantification accuracy, benchmarks show strong performance from StringTie3 [213] and Bambu [220] in mixture‐based evaluations, but also reveal that false novel splice junctions remain common across tools [41]. This underscores the importance of combining discovery‐oriented workflows with short‐read splice‐junction support and stringent filtering. When short‐ and long‐read data disagree on major isoforms, SG‐NEx supports using long‐read‐supported structures to define the expressed transcript set and then refining abundance estimates with short‐read depth [42]. In single‐cell and spatial long‐read transcriptomics, benchmarks further indicate that barcode/UMI detection and UMI error correction are major determinants of quantification accuracy [44]. For example, ONT's wf‐single‐cell (https://github.com/epi2me-labs/wf-single-cell) performs well in simulated datasets, whereas isoform‐centric methods can be informative for specific tasks but become more bias‐prone when UMI correction is inadequate. Thus, preprocessing quality is often as important as the downstream quantification method itself.

Finally, after transcript identification and before confident biological interpretation of quantification results, post‐processing QC is essential to ensure that the inferred transcriptome is supported by reliable alignments and is not dominated by technical artifacts (Figure 3G,H). This step is particularly important in long‐read analyses because apparent transcript novelty may arise not only from true alternative splicing, but also from RNA degradation, 5′ truncation, internal priming at A‐rich genomic regions, reverse transcriptase template switching, splice‐misalignment, and transcript‐collapse errors. Such artifacts are especially problematic when they generate false novel splice junctions or mis‐defined transcript ends, because these spurious models can enter the reference transcript set and directly distort downstream abundance estimation.

A useful conceptual framework is provided by SQANTI3 [223], which treats post‐processing QC as transcriptome curation rather than simple reporting. In this framework, isoforms are first classified into structural categories such as FSM, ISM, NIC, and NNC, and are then evaluated using transcript‐ and junction‐level descriptors, including splice‐site canonicity, short‐read junction support, chimerism, intra‐priming, reverse‐transcriptase‐switching signatures, and evidence supporting transcript ends from orthogonal resources such as Cap Analysis of Gene Expression (CAGE) [242], QuantSeq [243], and poly(A) motifs/sites. This category‐aware strategy is important because artifact risk is not uniform across transcript classes: reference‐matching isoforms and strongly supported alternative ends typically warrant different levels of confidence from transcript models that introduce unsupported donors/acceptors or poorly supported TSS/TTS.

As a practical guide, stringent post‐processing QC should therefore combine structural classification, junction‐centric filtering, and transcript‐end validation. For discovery‐oriented analyses, more permissive retention of candidate novel isoforms may be justified if they are explicitly labeled as provisional and prioritized for orthogonal validation. By contrast, for quantification‐focused analyses, filtering should be stricter, and transcript models with unsupported novel junctions, obvious intrapriming or reverse‐transcriptase‐switching signatures, or weakly supported 5′/3′ ends should be removed or downgraded before final quantification. AlignQC [118] summarizes alignment error profiles, whereas transcript‐structure QC tools such as SQANTI3 [223] and SQANTI‐reads [244] provide the evidence layers needed for this curation process. More recently, TUSCO [245] has been incorporated into the SQANTI3 ecosystem as an endogenous benchmarking framework that uses curated single‐isoform human and mouse genes as internal ground‐truth controls, enabling assessment of transcript reconstruction sensitivity, precision, and redundancy while capturing real‐sample biases such as RNA degradation, library effects, and insufficient depth. MultiQC [246] further facilitates consistent cross‐sample monitoring by integrating these outputs into a unified reporting framework.

### Identification and quantification of transposable element‐derived transcripts

TEs are mobile DNA sequences that have proliferated throughout genomes and collectively account for nearly half of the human genome. They are potential drivers of genome plasticity and can influence development, immunity, and disease through both insertional mutagenesis and diverse transcriptional outputs [17, 22, 247, 248] (Figure 5). Based on transposition mechanism, TEs are broadly grouped into Class I retrotransposons and Class II DNA transposons. Class I elements mobilize by a “copy‐and‐paste” route via an RNA intermediate and include long interspersed nuclear elements (LINEs) (e.g., L1), short interspersed nuclear elements (SINEs) (e.g., Alu), SVAs, and long terminal repeat (LTR) retrotransposons such as endogenous retroviruses (ERVs); Class II DNA transposons generally mobilize by a “cut‐and‐paste” mechanism [17] (Figure 5A).

**FIGURE 5:**
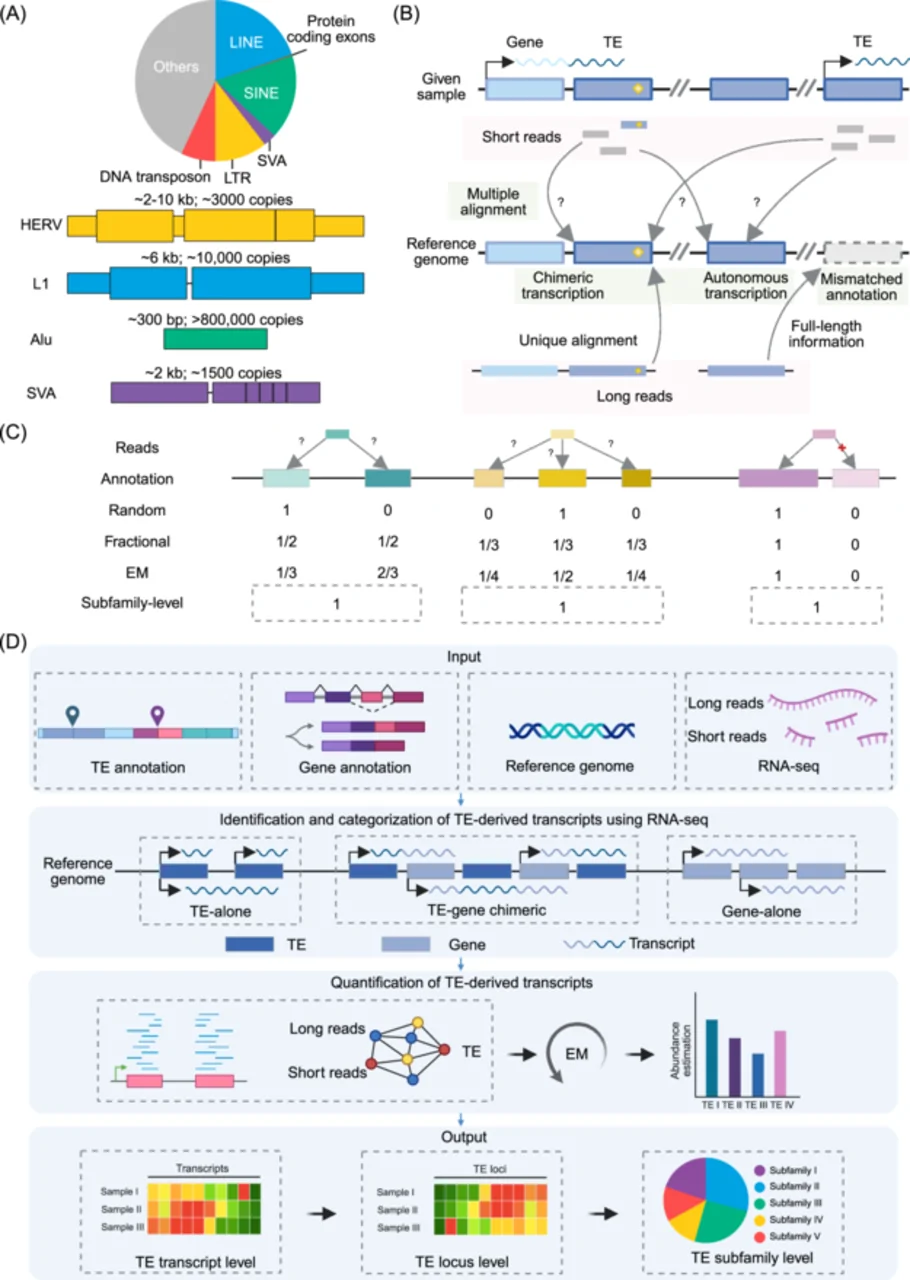
Characterization of transposable element‐derived transcripts. (A) Classification of TEs. Representative structural features of major TE families are illustrated. (B) Advantages of long reads over short reads for TE transcript analysis. Due to the repetitive nature of TEs, short reads frequently map to multiple genomic locations, resulting in ambiguous alignments and difficulty in distinguishing between autonomous TE transcription and gene‐TE chimeric transcripts. In contrast, long reads can span larger portions of transcripts, enabling unique alignment and improved resolution of TE‐derived transcriptional events. (C) Schematic representation of traditional TE quantification strategies based on RepeatMasker annotations. Conventional TE quantification methods rely on RepeatMasker TE annotations and use different algorithms to assign reads mapped to repetitive TE loci. These approaches include unique assignment, fractional assignment, and expectation‐maximization (EM)‐based probabilistic models to distribute multi‐mapping reads among candidate TE loci or subfamilies. (D) Overview of the LongTEQuant pipeline for transcript‐level TE quantification. The LongTEQuant workflow integrates TE annotations, gene annotations, a reference genome, and RNA‐seq data (long‐read or short‐read) as inputs. TE‐derived transcripts are first identified and categorized at the transcript level into TE‐alone transcripts, TE‐gene chimeric transcripts, and gene‐only transcripts. Quantification is then performed using long‐read, short‐read, or both types of data to estimate TE expression across transcripts. The final outputs include TE expression profiles summarized at multiple levels, including TE subfamily, TE locus, and TE‐derived transcript levels. EM, expectation‐maximization; LTR, long terminal repeat; LINE, long interspersed nuclear element; SINE, short interspersed nuclear element; ORF, open reading frame; RNA‐seq, RNA sequencing; TE, transposable element; TIR, terminal inverted repeat; UTR, untranslated region. Created in BioRender. Li, J. (2026) https://BioRender.com/yfm6pj3.

TE expression is usually quantified at either the subfamily level (aggregating signal across many genomic copies) or the locus level (assigning expression to specific insertions). Achieving accurate TE quantification is difficult for three fundamental reasons (Figure 5B). First, TE insertions are often polymorphic, so reference annotations may not match the insertions present in a given sample. Second, high hierarchical sequence similarity within and across TE families causes pervasive multi‐mapping, limiting unique read assignment and reducing locus resolution. Third, TE sequences frequently occur within host‐gene transcripts or within TE‐gene chimeric transcripts created by exonization, alternative promoters, readthrough, or splicing, which makes it hard to separate autonomous TE transcription from TE fragments carried within gene isoforms [34, 249]. These challenges have motivated a toolbox ecosystem that can be organized by the core principle used to resolve ambiguity (Figure 6).

**FIGURE 6:**
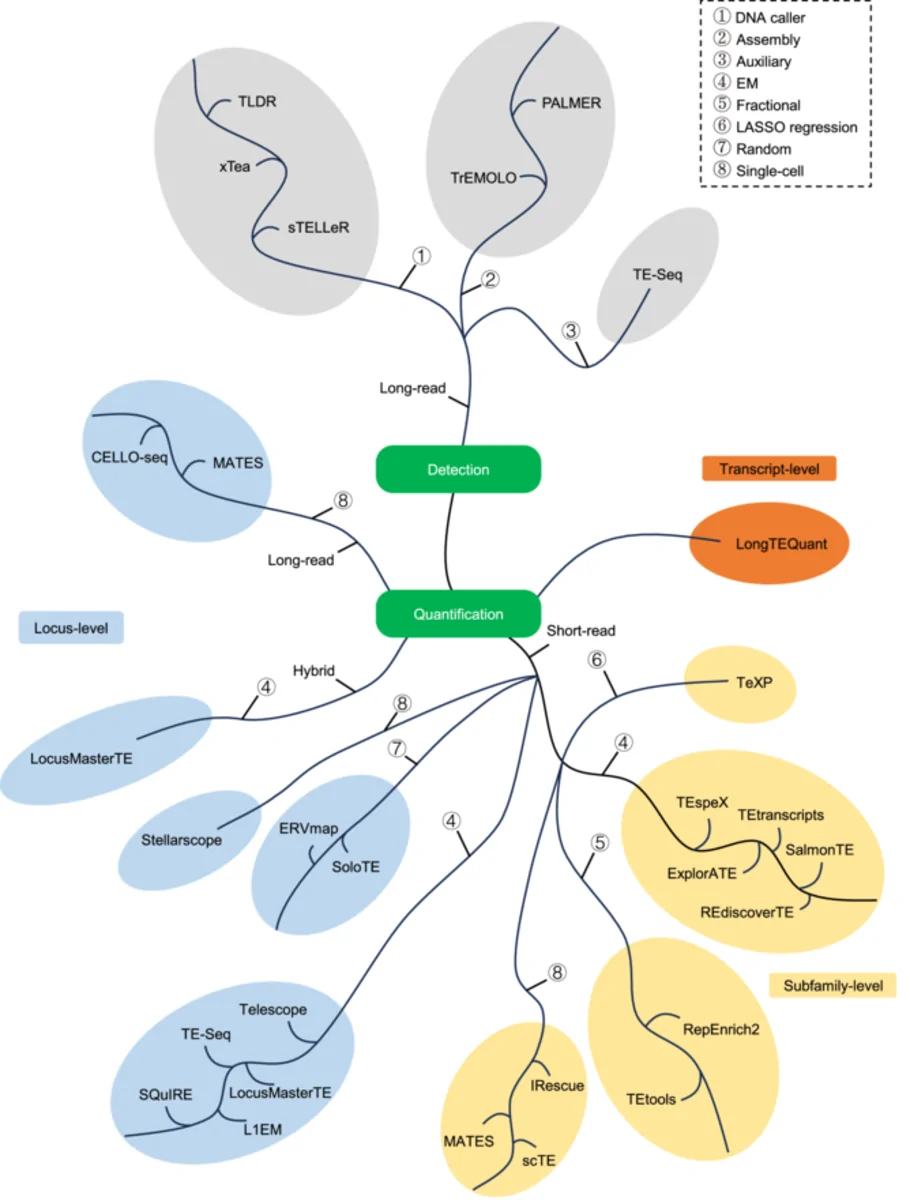
Computational landscape for TE insertion detection and TE‐derived transcript quantification. This diagram organizes representative computational strategies for TE detection and expression quantification according to sequencing data type, quantification resolution, and core algorithmic principle. The detection branch summarizes long‐read‐based methods classified as (1) DNA callers, (2) assembly‐based methods, and (3) auxiliary approaches. The quantification branch is divided into three main data regimes: short‐read, long‐read, and hybrid approaches. Short‐read‐based TE quantification methods are further grouped by resolution, including subfamily‐level and locus‐level approaches. Methods are also categorized according to their core computational strategies, indicated by numbers along the branches: (4) EM, (5) fractional assignment, (6) LASSO regression, (7) random assignment, and (8) single‐cell‐oriented approaches. Long‐read‐based methods leverage long‐range transcript information to improve mapping specificity and TE expression inference, whereas hybrid methods integrate short‐ and long‐read data to enhance locus‐level resolution and quantification accuracy. In addition, transcript‐level frameworks directly quantify TE‐derived transcripts using transcript‐resolved information. Tool names are positioned within each cluster to illustrate representative implementations under each methodological category. EM, expectation‐maximization; LASSO, least absolute shrinkage and selection operator; TE, transposable element.

Methods addressing the first challenge focus on building a sample‐specific TE landscape by detecting non‐reference insertions before quantifying TE expression. Long‐read‐based TE insertion callers such as TLDR [250], xTea [251], and sTELLeR [252] typically align reads to the reference genome and TE consensus libraries (often using minimap2 [202]) to infer breakpoints. They are generally more sensitive to complex insertions and map better in repetitive regions than short‐read approaches, but performance depends on sequencing depth, error profiles, TE type, and genomic context. Assembly‐based tools such as PALMER [253] and TrEMOLO [254] add local assembly to recover more complete insertion sequences, improving structural resolution at the cost of higher computational requirements. Auxiliary approaches (e.g., TE‐Seq [255] when supported by long‐read genome sequencing data) can further recover missing reference elements and detect active non‐reference insertions, although they may be limited by assay design and incomplete coverage of insertion diversity.

Methods targeting the second challenge aim to resolve ambiguity from multi‐mapping, particularly in short‐read RNA‐seq (Figure 5C). Subfamily‐level quantifiers (*e.g*., TEspeX [256], TEtranscripts [257], SalmonTE [258], ExplorATE [259], REdiscoverTE [260], TEtools [261], RepEnrich2 (https://github.com/nerettilab/RepEnrich2), TeXP [262], scTE [263], IRescue [264]) often use EM or probabilistic assignment to estimate aggregate abundance without locating the source insertion; they are efficient and effective for detecting broad TE activation but cannot reliably identify which copies are expressed. Locus‐level methods (e.g., TE‐Seq [255], MATES [265], SQuIRE [266], Telescope [267], LocusMasterTE [268], L1EM [269], SoloTE [270], ERVmap [271], Stellarscope [272], CELLO‐seq [273]) attempt to distribute reads across candidate loci using models of read generation and mapping uncertainty; they can provide site‐resolved signals when unique sequence is available in or near a TE, but are sensitive to model assumptions and local mappability. Recent hybrid strategies use long reads to improve short‐read assignment. For example, LocusMasterTE [268] incorporates long‐read‐derived TPM priors into an EM framework to guide reassignment of multi‐mapped short reads, which often stabilizes estimates but requires matched datasets and careful normalization across technologies. In single‐cell settings, CELLO‐seq [273] reduces ambiguity experimentally by using long UMIs and cell barcodes for error correction and molecule counting, whereas MATES [265] uses flanking unique context and deep learning to probabilistically assign multi‐mapped reads; both can improve locus assignment, but depend on protocol constraints, training generalization, and the availability of informative context. Related locus‐centric genotyping concepts (*e.g*., adaptations of T‐lex2 [274]) provide additional options but may inherit limitations from their original assumptions [275].

Methods addressing the third challenge focus on defining TE‐derived transcripts in a transcript‐structured manner. This problem is exacerbated by the mismatch between TE annotations, which are largely interval‐ and class‐based (e.g., RepeatMasker), and gene annotations, which are transcript‐based and encode TSSs, splice junctions, poly(A) sites, and strand. As a result, TE quantification can be inflated by readthrough or host‐gene transcription, and TE‐gene chimeras can be difficult to identify systematically. Integrative workflows such as LongTEQuant (https://github.com/gaoshiwen/LongTEQuant) (Figure 5D) mitigate these issues by reconstructing transcripts from RNA‐seq (short or long reads), integrating transcript models with TE annotations, and classifying transcripts into TE‐alone, TE‐gene chimeric, and Gene‐alone categories prior to quantification. The main benefit is improved biological interpretability because quantification is performed on explicit transcript entities rather than on repeat intervals. The main limitation is reliance on transcript reconstruction quality (sequencing depth, error rates, algorithm choice) and on robust classification criteria for mixed transcripts. In practice, integrating widely used transcript identification and quantification tools (e.g., IsoQuant [214], Bambu [220], FLAIR [212], StringTie3 [213], miniQuant [141], Salmon [234], RSEM [233], and kallisto [232]) can reduce errors, but does not remove these dependencies.

Long‐read transcriptomics is increasingly central to TE quantification because it captures full‐length molecules, which directly improves both locus assignment and chimeric transcript resolution. Long reads often span TE bodies together with unique flanking sequence, splice junctions, and transcript ends, enabling clearer isoform delineation and more confident identification of TE‐initiated transcripts, TE‐mediated APA, TE‐driven alternative splicing, and TE‐gene chimeras. A practical strategy is therefore to use long‐read RNA‐seq (cDNA and/or direct RNA) to build transcript‐level TE annotations that include TSSs, splicing patterns, poly(A) sites, and strand, integrate these annotations with sample‐specific insertion maps when available, and quantify expression at the finest feasible resolution, ideally as TE‐derived transcript isoforms assigned to specific loci. Subfamily‐level estimates remain useful summaries, but are most informative when derived by aggregating well‐defined locus‐ and transcript‐resolved results.

## TRANSCRIPTOME COMPLEXITY: NOVEL INSIGHTS FROM LONG READS

After transcript reconstruction, LRS enables a more direct and comprehensive view of transcriptome complexity. Because single reads preserve long‐range exon connectivity and, in DRS, retain native strand information and terminal features, long reads are particularly powerful for resolving alternative splicing, ATSS, APA, poly(A) tail profiling, circular RNAs, fusion transcripts, and ASE in a unified isoform‐centric framework (Table S3).

### Alternative splicing

Alternative splicing generates multiple RNA isoforms from a gene and is a major driver of proteomic diversity [5] (Figure 7A). Long reads can span multiple exons in a single molecule, allowing exon connectivity and coordinated splicing patterns to be observed directly rather than inferred from fragmented short reads [276]. DRS preserves native RNA features, making it possible to interpret splicing together with poly(A)‐related signals and other regulatory layers (*e.g*., RNA modifications) on the same molecule [38, 277, 278].

**FIGURE 7:**
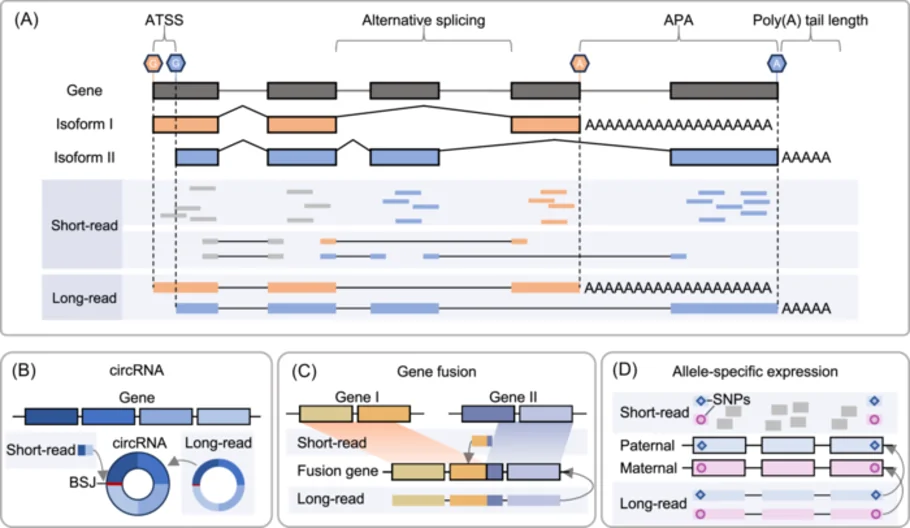
Advantages of long reads over short reads in resolving complex transcription events. (A) Long reads can identify distinct 5' and 3' transcript ends and accurately determine which isoform a read originates from, whereas short reads often have difficulty with precise mapping. Long‐read sequencing can also resolve complex alternative splicing events with high confidence, whereas short reads frequently suffer from ambiguous mapping because many reads (gray) originate from regions shared by multiple isoforms, while only isoform‐specific reads (blue and orange) can be uniquely assigned. In addition, long reads can directly measure the length of the poly(A) tail, whereas short reads typically cannot reliably determine long poly(A) tail lengths. (B) Long reads provide stronger global resolution of full‐length circular RNA structures compared with short reads. (C) Long reads can directly capture the full‐length structure of fusion transcripts, while short reads usually provide only partial or local information. (D) Long reads can accurately determine whether a full‐length transcript originates from the paternal or maternal allele through linking multiple SNPs, whereas short reads can only assign parental origin for the limited subset of reads containing informative SNPs. ATSS, alternative transcription start site; APA, alternative polyadenylation; BSJ, back‐splice junction; circRNA, circular RNA; SNP, single‐nucleotide polymorphism.

Splicing analysis typically follows two complementary routes. Event‐level approaches (e.g., rMATS‐turbo [279], IRFinder‐S [280], DIGGER [281], Exon Ontology [282], NEASE [283], DoChaP [284]) quantify canonical events such as exon skipping, alternative 5′ and 3′ splice sites, intron retention, and mutually exclusive exons, often using percent spliced in (PSI) like metrics and replicate‐aware statistics; rMATS‐turbo [279] is widely used for differential splicing at this level, while IRFinder‐S [280] is tailored for high‐precision intron retention quantification. In addition, graph‐based frameworks such as MAJIQ [285, 286] extend this paradigm by modeling local splicing variations (LSVs), enabling the quantification of both classical event types and more complex, non‐binary splicing patterns. Isoform‐centric approaches (e.g., MISO [287], IUTA [288], DRIMSeq [289], satuRn [290], IsoformSwitchAnalyzeR [291], tappAS [292]) treat full‐length transcripts as the primary unit, better capturing combinations of multiple events and their functional consequences; tools such as IsoformSwitchAnalyzeR [291] and tappAS [292] integrate open reading frame (ORF) prediction, domain changes, and nonsense‐mediated decay (NMD) sensitivity. In long‐read workflows, generating reliable full‐length isoform models is the critical first step, commonly using callers such as IsoQuant [214], Bambu [220], FLAIR [212], and StringTie3 [213], after which event‐level summaries can be added when classical event statistics are needed.

### Alternative transcription start site

ATSS usage increases isoform diversity by producing distinct 5' ends associated with different promoter choice, first exon usage, and untranslated region sequence. This can in turn reshape RNA stability, translation, coding potential, and regulatory element composition [293, 294, 295] (Figure 7A). Short‐read strategies such as CAGE provide strong promoter‐level evidence but often cannot connect a specific start site to the full transcript body, making it difficult to determine which splicing patterns and 3' ends are coupled to a given promoter at alternatively‐spliced loci [242, 296, 297, 298].

Long reads address this linkage problem by preserving continuity from the 5' region through internal exon structure to the 3' end, enabling promoter switching and alternative first‐exon usage to be interpreted together. In practice, ATSS analysis is best embedded in transcript reconstruction and boundary refinement workflows using tools such as FLAIR [212], StringTie3 [213], IsoQuant [214], and Bambu [220] to collapse reads into transcript models and quantify isoform abundance. Because long‐read RNA‐seq datasets can suffer from incomplete 5' capture and systematic truncation, read‐start ends/coordinates of read alignment alone are often insufficient for de novo TSS discovery, so start‐site clustering methods such as NAGATA [299] and LoRTIA (https://github.com/zsolt-balazs/LoRTIA) and refinement strategies that integrate annotations or external 5'‐end datasets are commonly used, with cap‐associated evidence like CAGE [242] and ReCappable‐seq [297] providing high‐confidence validation when precise TSS catalogs are required.

### Alternative polyadenylation

APA diversifies the transcriptome by generating distinct 3' ends, which alters 3' UTR length and cis‐regulatory elements, and influences RNA localization, stability, and coding potential [300, 301, 302] (Figure 7A). Short‐read RNA‐seq and dedicated 3'‐end methods can detect global shifts in poly(A) site usage, but they often struggle to assign a specific cleavage site to a particular full‐length isoform or to resolve coupling between APA, upstream splicing, and promoter choice [303, 304, 305].

LRS facilitates the detection of poly(A) sites and their linkage to full‐length isoform structures. APA can be characterized either by clustering read ends to build poly(A) site catalogs and quantify differential usage, as supported by tools such as APALORD [306] and LAPA [307], or by first reconstructing high‐confidence isoforms with long‐read transcriptome tools such as FLAIR [212], IsoQuant [214], StringTie3 [213], or Bambu [220] and then performing gene‐wise 3'‐end clustering within each isoform set to study coordinated RNA processing. Given technical artifacts such as internal priming and end‐alignment uncertainty, robust analyses typically combine end‐site clustering with stringent filtering, replicate support, and conservative interpretation of weakly supported sites, selecting direct end‐site discovery when site cataloging is the primary goal and isoform‐aware inference when mechanistic coupling across transcript features is the focus.

### Poly(A) tail length

Poly(A) tails are dynamic regulators of mRNA maturation, stability, and translation [308, 309], with effects that depend on cellular context and tail composition (Figure 7A). Conventional assays such as TAIL‐seq [310] provide valuable transcriptome‐wide measurements but generally separate tail information from the full‐length isoform context, limiting the ability to associate tail length with specific isoforms, splicing patterns, cleavage choices, or RNA modification states [95].

DRS enables tail‐length estimation on the same molecule that carries the transcript sequence by detecting the characteristic poly(A) signal region and converting its duration to nucleotides using read‐specific translocation rates [38, 113, 311]. Practical workflows often use established callers such as Nanopolish (https://github.com/jts/nanopolish) and tailfindr [311], while Dorado (https://github.com/nanoporetech/dorado) can provide built‐in poly(A) or poly(T) estimates directly in BAM outputs for streamlined processing. Because tail‐length estimates are sensitive to signal noise, speed variation, degradation, and chemistry, analyses typically emphasize replicate‐supported distribution shifts and relative differences across conditions or isoforms rather than relying on absolute nucleotide‐level values, integrating tail profiles with reconstructed isoforms and APA patterns to obtain the most informative isoform‐resolved view of 3′‐end regulation [85, 95, 312, 313].

Beyond homopolymeric adenosines, emerging evidence has revealed that poly(A) tails frequently contain non‐adenosine residues (*i.e*., C, G, U) interspersed within the adenosine stretch [97, 99, 314]. DRS is uniquely suited to study such composite tails, while detecting non‐adenosines within homopolymeric stretches remains challenging due to the uniform electrical signals of consecutive identical bases. To address this, Ninetails [315], a neural network‐based method, was developed to accurately identify and classify individual C, G, and U residues within poly(A) tails from DRS data.

### Circular RNA

Circular RNAs (circRNAs) arise from back‐splicing to form covalently closed molecules and often exhibit complex internal splicing that yields multiple circRNA isoforms [316] (Figure 7B). Short‐read methods largely rely on detecting back‐splice junctions, which makes full‐length reconstruction difficult when circRNA sequences closely resemble linear transcripts [317, 318, 319]. Long reads can span entire circRNA molecules and therefore provide a stronger basis for defining circRNA architecture and isoform diversity [320].

Long‐read circRNA sequencing typically enriches circRNAs through rRNA depletion, RNase R digestion, and poly(A)‐negative selection, followed by library construction using rolling‐circle RT or amplification strategies, with protocols such as CIRI‐long [321, 322], circFL‐seq [323], and isoCirc [324] widely used, and circNick‐LRS [325] offering an alternative via selective cleavage to produce linear templates. Identification generally combines back‐splice junction detection with full‐length reconstruction, and because sensitivity can vary substantially across tools with limited overlap between call sets, combining callers is often used to improve robustness [326]. Quantification remains challenging with long reads due to low circRNA fractions and concatemer‐related artifacts, so expression estimation is frequently anchored by short‐read‐based approaches such as CIRIquant [327], while long‐read experiments benefit from synthetic circRNA spike‐ins for normalization and careful consideration of RNase R‐associated biases [328].

### Fusion gene

Gene fusions are widespread in cancer and can result from read‐through transcription, structural rearrangements, TE‐mediated events, and rare trans‐splicing (Figure 7C) [329, 330]. Long reads improve fusion discovery by capturing breakpoint‐spanning molecules and full exon connectivity, enabling more confident fusion partner assignment and better isoform resolution than short‐read approaches [331].

Bulk fusion detection often begins with splice‐aware genome alignment followed by chimeric read identification, as implemented in tools such as LongGF [332] and JAFFAL [331], with auxiliary annotation support from frameworks like SQANTI3 [223]. To reduce false positives, clustering and statistical filtering strategies are commonly applied, including Genion [333] and FusionSeeker [334], while reconstruction‐and‐realignment approaches such as FUGAREC [335], CTAT‐LR‐fusion [24], and FLAIR‐fusion [336], strengthen breakpoint and splice‐structure support and enable isoform‐level fusion modeling. For higher robustness, multi‐caller integration methods such as GFvoter [337] can combine complementary strengths, and for single‐cell contexts, tools designed for sparse and heterogeneous data, such as GFHunter [338] and LongFUSE [339], are often preferred, with IFDlong [340] providing a route to transcript‐level fusion quantification using an EM‐like framework after detection.

### Allele‐specific expression

ASE and splicing analyses benefit from long reads because single molecules can span multiple heterozygous variants, reducing mapping ambiguity and enabling direct phasing of isoforms onto haplotypes [341] (Figure 7D). Compared with short reads, this supports more reliable separation of allele‐specific effects on expression level and splice choice, and it helps disentangle cis‐ and trans‐ contributions to expression and splicing regulation [342].

Effective ASE workflows typically focus on reducing reference‐mapping bias and achieving haplotype‐aware quantification. Recent advances in long‐read RNA‐seq have enabled unified frameworks for SNP calling, haplotype phasing, and allele‐specific analysis directly from transcriptomic data, as exemplified by longcallR [341], which leverages full‐length reads to jointly resolve variants, haplotypes, and ASE. Personalized haplotype references, as used by LORALS [343], improve allele assignment and support both ASE and splicing, while variation‐graph‐based approaches such as RPVG [344] connect pangenome representations with haplotype‐specific transcript inference. Event‐level strategies such as isoLASER [345] combine local reassembly with haplotype clustering to dissect allele‐associated splicing effects within individuals, and isoform‐centric pipelines such as FLAIR2 [346] extend long‐read transcript annotation to haplotype‐aware analysis. For studies emphasizing haplotype‐resolved isoform reconstruction and parent‐of‐origin effects, tools such as IDP‐ASE [347], HapIso [348], and IsoPhase [349] provide complementary capabilities across diverse organisms and experimental designs.

## DOWNSTREAM ANALYSES: FROM TRANSCRIPTS TO BIOLOGICAL INSIGHTS

Long reads shift transcriptomics from fragment‐based inference to direct, single‐molecule observation of full‐length transcripts. Because a single read can capture complete splice connectivity together with transcript boundaries (TSS/TTS) and poly(A) site choice, it can enable more confident isoform definition and reduce short‐read attribution errors caused by shared exons, incomplete annotations, and disconnected end‐splicing information [18]. Long reads can link multiple heterozygous variants to support allele/haplotype phasing and span fusion breakpoints to resolve chimeric transcript structures [341]. Together, these strengths are driving downstream analysis beyond traditional gene‐level differential expression toward a transcript‐centric framework that integrates structure, quantification, function, and regulation, covering differential transcript expression (DTE), differential splicing, isoform switching, isoform‐level regulatory network inference, function prediction, and visualization (Figure 8).

**FIGURE 8:**
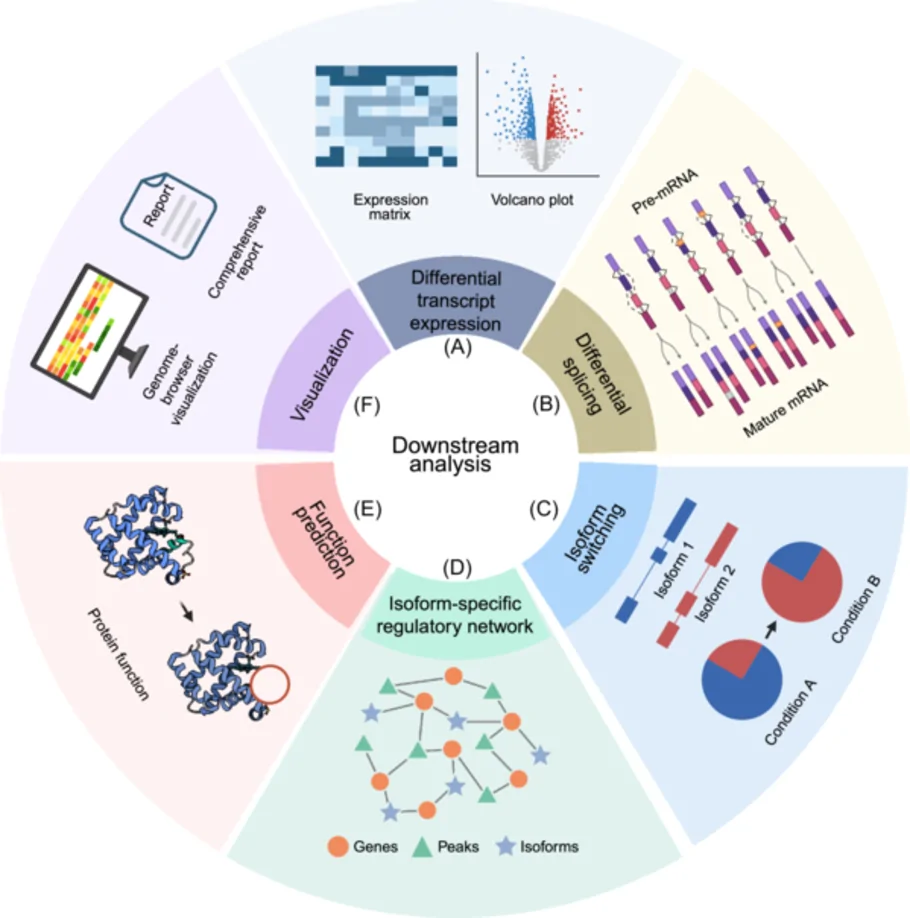
Downstream analyses for transcriptomics studies enabled by LRS. (A) Differential transcript expression analysis generates an expression matrix and identifies significantly changed isoforms between conditions. (B) Differential splicing analysis detects condition‐associated alternative splicing events with a full‐length picture. (C) Isoform switching analysis quantifies changes in the relative usage of isoforms from the same gene across conditions. (D) Isoform‐specific regulatory network inference links transcript isoforms to upstream regulators and downstream targets to reveal isoform‐level regulatory programs. (E) Functional prediction annotates isoforms and evaluates potential impacts on protein domains and biological functions. (F) Visualization and reporting integrate results through genome browsers and comprehensive reports to facilitate interpretation and dissemination. Created in BioRender. Li, J. (2026) https://BioRender.com/14i3g8k.

### Differential transcript expression

Differential transcript expression (DTE) tests whether the absolute abundance of specific transcript isoforms differs across conditions and is the primary goal of isoform‐resolved quantification (Figure 8A). Because isoforms can vary in coding sequence and regulatory features, isoform‐level changes can drive phenotypic diversity, cell‐state transitions, and development [350, 351]. Clinically, pathogenic isoform reproportioning may occur without a change in total gene expression; for example, cancer progression and drug resistance have been associated with the overexpression of specific oncogenic isoforms, making DTE valuable for biomarker discovery and therapeutic targeting [352].

Methodologically, DTE workflows can be grouped by the data used for isoform quantification. For short‐read RNA‐seq, isoform abundance is typically inferred by probabilistic assignment of fragments to transcripts, using tools such as Salmon [234], kallisto [232], and RSEM [233], often followed by uncertainty‐aware DTE testing with sleuth [353] or count‐based testing after transcript‐level summarization with tximport [354] and models such as DESeq2 [355], edgeR [356], or limma‐voom [357]. This strategy benefits from the high sequencing depth usually affordable by short reads and mature statistical models, but it is vulnerable to read misassignment when isoforms share exons, transcript ends are heterogeneous, or annotations are incomplete, which can bias abundance estimates and inflate false positives, particularly for complex loci. For long‐read RNA‐seq, isoforms can be directly observed and quantified with IsoQuant [214], FLAIR [212], Bambu [220], StringTie3 (long‐read mode) [213], miniQuant [141], and then tested using the same differential‐expression backends (DESeq2 [355], edgeR v4 [358], limma‐voom [357]) or long‐read‐oriented integrated frameworks such as IsoTools 2.0 [215]. Long reads reduce ambiguity in isoform definition and improve TSS/TTS completeness, but long‐read‐only DTE can be constrained by lower effective sequencing depth and platform‐ or protocol‐specific biases, increasing the importance of normalization, replicate number, and filtering. A third, increasingly adopted category is hybrid analysis: long reads are used to build or curate a high‐confidence, study‐specific transcript reference, while high‐depth short reads provide precise quantification against that reference (e.g., Salmon [234], kallisto [232], or RSEM [233] with tximport [354], then DESeq2 [355], edgeR [356], or limma‐voom [358]), combining structural accuracy with statistical power; its main limitation is the added complexity of reference harmonization and cross‐platform bias control.

For long‐read transcriptomics, a practical recommendation is to use long reads to construct or refine a sample‐relevant isoform annotation, quantify at the transcript level against this fixed reference, and apply established differential‐expression models matched to the experimental design. DESeq2 [355] and edgeR v4 [358] are robust defaults for count‐based testing, especially with small to moderate sample sizes, while limma‐voom is advantageous for multi‐factor designs with covariates and interactions; for abundance estimates where quantification uncertainty should be carried through, sleuth [353] is a suitable option, and linear mixed models are preferred for repeated‐measures or hierarchical designs. In long‐read‐dominant studies that benefit from a single structure‐aware workflow, IsoTools 2.0 [215] can integrate isoform discovery, quantification, and differential analyses.

In all cases, long‐read DTE should explicitly address library type, read‐length distribution, 3' bias, PCR duplicates, platform‐specific error profiles, and isoform effective‐length comparability, and results should be reported with log2 fold changes and confidence intervals, multiple‐testing adjustment, and consistency checks between gene‐ and isoform‐level signals. Key findings are best supported by orthogonal validation such as isoform‐specific qPCR, targeted LRS, and proteomics or Ribo‐seq when coding changes are implicated.

### Differential splicing

Differential splicing (DS) tests whether the configuration of RNA splicing changes between conditions at a local, event‐like level, capturing patterns such as exon skipping, mutually exclusive exons, alternative splice‐site choice, and intron retention (Figure 8B). Unlike analyses that focus only on changes in whole‐isoform abundance, DS is tightly linked to cis‐regulatory elements and trans‐acting regulators, including splicing factors and RNA‐binding proteins, which makes it particularly useful for mechanistic interpretation and regulator prioritization [359]. Because alternative splicing expands proteomic and regulatory diversity, aberrant DS is a common feature of genetic disorders and cancer, where disruptions in the splicing machinery can lead to widespread mis‐splicing [360]. Additionally, recent studies have linked splicing dysregulation to aging and age‐associated decline, where disruptions in RNA‐binding proteins and spliceosomal components lead to widespread mis‐splicing [361, 362, 363, 364].

Current DS methods can be organized by the data type and the way splicing is represented and quantified. Short‐read RNA‐seq remains widely used and typically relies on junction and exon‐intron evidence, but it cannot reliably phase distant events into full transcript configurations and is sensitive to annotation incompleteness and structural ambiguity, which can reduce power for complex or non‐canonical events [365, 366]. Within short‐read‐driven approaches, event‐template methods such as rMATS [367] provide standardized outputs for common event classes and are straightforward to interpret, but they are less flexible for multi‐branch or complex splicing. Graph/probabilistic local‐variation models such as MAJIQ [285, 286] are well‐suited to multi‐branch splicing and quantify uncertainty, at the cost of more complex modeling and interpretation. Isoform‐abundance‐derived event methods such as SUPPA2 [368] compute PSI from transcript quantification, making them convenient when reliable isoform estimation and annotations exist, but they inherit biases from transcript inference and annotation quality. Annotation‐light intron‐clustering methods such as LeafCutter [369] reduce reliance on transcript models and can discover novel differential intron usage, although mapping clusters back to canonical event labels or biological interpretation can be less direct. Count‐based exon/junction frameworks such as DEXSeq [370] and JunctionSeq [371] provide robust generalized linear‐model testing and serve as strong baselines, but their features do not always correspond cleanly to discrete splicing events and can be harder to translate into specific splice patterns.

In practice, a recommendation is to use long reads to establish high‐confidence isoform models and event boundaries, then choose a quantification and testing strategy that matches the study's depth and design. When read depth is limited, a common and effective hybrid approach is to define the event space with long reads and quantify events with higher‐coverage data (often short reads) to obtain stable PSI and ΔPSI estimates with controlled FDR; tools such as rMATS [367] (and long‐read‐adapted variants like rMATS‐long (https://github.com/Xinglab/rMATS-long)), MAJIQ [285, 286], LeafCutter [369], DEXSeq [370], or JunctionSeq [371] can then be applied using the LRS‐informed annotation or intron sets to reduce systematic biases. When long‐read coverage is sufficient and an integrated, structure‐aware workflow is preferred, long‐read‐centric suites such as IsoTools 2.0 [215] can perform event‐level DS and related isoform‐usage analyses within a single framework, improving consistency between discovery, quantification, and reporting.

### Isoform switching

Isoform switching refers to a condition‐dependent change in the dominant isoform of a gene, or a reversal in usage between a key isoform pair (Figure 8C). It is useful because switching often implies functional change: it can alter coding‐sequence completeness, reading frame, and the proportion of nonsense‐mediated decay candidates, reshape domains, signal peptides, and transmembrane segments, and rewire 5'/3' UTR features such as RBP/miRNA sites, upstream open reading frames (uORFs), and poly(A) signals [372]. In biomedical settings, isoform switching analysis helps prioritize mechanistically interpretable candidates and can support clinically oriented questions such as identifying recurrent, potentially targetable isoform‐derived neoantigens across tumors [373].

Methodologically, isoform switching can be inferred from short‐read quantification, long‐read isoform‐resolved measurements, or hybrid designs, and it is important to distinguish a statistically significant switch from a switch with credible functional consequences. Short‐read workflows typically quantify transcripts with Salmon [234], kallisto [232], and RSEM [233] and then test differential transcript usage, ranking candidates by effect size (e.g., changes in isoform usage fraction) [291, 374]. They are cost‐effective and powerful with sufficient depth, but are vulnerable to incomplete annotations and ambiguous read assignment among similar isoforms, especially near transcript ends, which weakens ORF/NMD inference and can generate artifactual switches. Long reads directly capture full‐length structures and reduce end ambiguity, improving confidence in ORF and consequence predictions, but quantification can be limited by lower depth and platform‐ or protocol‐specific biases. For consequence‐focused interpretation, IsoformSwitchAnalyzeR [291] provides an integrated workflow for switch calling, consequence annotation (including ORF/NMD), and enrichment analysis, with optional links to Pfam [375], InterPro [376], SignalP [377], and TMHMM [378]; tappAS [292] emphasizes functional‐feature usage with visualization and statistics; and Biosurfer [379] helps track transcript‐to‐protein differences to better triage candidates, typically as a complement to formal testing.

Building on these considerations, long‐read transcriptomics is best used to create a high‐confidence, sample‐specific isoform catalog with accurate transcript ends, after which isoform usage should be quantified using a strategy matched to coverage and design. When long‐read depth is modest, a practical hybrid approach is to use long reads for discovery and validation, quantify isoform usage with deep short reads against the curated reference, and call switching based on both statistical significance and effect size. With sufficient long‐read coverage, long‐read quantification can be used directly, provided normalization, duplicate handling, and platform bias are carefully controlled.

### Isoform‐specific regulatory network

Isoform‐specific regulatory networks move beyond gene‐level graphs by treating transcript isoforms as separate nodes with distinct structures and regulatory elements (Figure 8D). This is important because splicing can alter protein interaction surfaces and localization signals, while alternative 5'/3' UTRs can change miRNA and RBP binding landscapes, producing different or even opposite outcomes from the same gene locus [380]. Short‐read gene‐level networks often mask these effects by averaging isoform signals and by poorly linking UTR features to the matching coding sequence, limiting mechanistic interpretation of post‐transcriptional regulation. LRS helps by resolving full‐length isoforms and assigning exons, UTRs, and poly(A) sites to specific transcripts, providing a clearer substrate for network inference [214, 241, 342].

Methods mainly differ in how they define edges and what evidence supports them. Co‐expression/co‐variation approaches infer edges from correlated isoform abundance; WGCNA [381] builds interpretable modules and hubs, and MEGENA [382] can capture nested, multiscale modules when the feature set is large. They are straightforward but sensitive to confounding and dimensionality, require stability checks, and correlation does not imply direction. Sequence‐ and element‐driven approaches predict miRNA/RBP regulation from isoform‐resolved UTRs, using tools such as TargetScan (miRNAs) [383] and RBPmap (RBPs) [384]; they scale well and are mechanistically motivated, but can be noisy and typically need empirical binding support from eCLIP [385] (with peak callers such as PureCLIP [386]) and functional readouts such as Ribo‐seq, proteomics, or perturbations. Multi‐omics integration anchors isoform‐level regulation with orthogonal signals, especially cis QTLs (sQTLs, apaQTLs) together with chromatin or promoter activity; QTLtools [387] is commonly used for mapping and coloc [388] for colocalization. Curated resources such as RegNetwork 2025 [389] are best used for benchmarking and annotation, while keeping predicted edges separate from evidence‐supported ones.

For long‐read transcriptomics, a practical workflow is to first build a high‐confidence, sample‐matched isoform catalog with accurate UTR and poly(A) annotations, then infer networks in layers that separate prediction from support. Use long reads to define isoform nodes and regulatory elements, and use deeper short reads (or sufficiently deep long reads) to stabilize isoform quantification for co‐variation analyses, with robustness assessed across resampling, batches, and cohorts. Compute miRNA/RBP edges on isoform‐specific UTRs and filter or weight them using eCLIP [385] and downstream functional evidence, reserving perturbation experiments for key hubs and modules. When genotype data are available, add sQTL/apaQTL and colocalization to prioritize cis‐linked edges and reduce gene‐level masking. In reporting, clearly distinguish correlation modules, sequence‐based predictions, and edges supported by binding or genetic evidence, emphasizing results that replicate across datasets and evidence types.

### Function prediction

Function prediction in isoform‐resolved transcriptomics assigns each transcript variant plausible molecular consequences and testable mechanisms, linking isoform changes to protein output, localization, stability, and regulatory sensitivity (Figure 8E). It aims to explain how a specific isoform alters function, for example through domain gain or loss, changes in interaction interfaces, signal peptides, or transmembrane segments, UTR rewiring that affects translation, or increased NMD that reduces protein levels [380, 390, 391]. This step connects differential isoform signals to concrete hypotheses and helps prioritize candidates for validation.

Methods vary by input data and evidentiary strength. Transcript‐sequence‐based consequence annotation infers coding potential, ORFs, frame preservation, premature termination, NMD propensity, and protein features such as domains, signal peptides, and transmembrane regions; IsoformSwitchAnalyzeR [291] and tappAS [292] are widely used to run these annotations at scale and relate them to isoform switching or differential usage. These approaches are fast and interpretable, but they depend on accurate isoform models and transcript ends; fragmented or misassembled short‐read reconstructions can yield chimeras and false ORFs, producing misleading feature calls. Proteoform‐focused tools such as Biosurfer [379] compare predicted impacts across transcript, codon, and protein representations and help attribute changes to alternative TSS, splicing, or termination, but their conclusions remain predictive without protein‐level support. Structure‐oriented analyses add a mechanistic check on whether splicing changes likely disrupt folded regions or interaction interfaces, yet they are typically best suited as a targeted layer for high‐priority events and can be limited by structural coverage and context.

With long‐read transcriptomics, full‐length isoform resolution should be the starting point, followed by staged, evidence‐aware interpretation. Build a high‐confidence, sample‐matched isoform set with accurate ends and sufficient expression support, then annotate ORFs and consequence classes (frame disruption, truncation, NMD likelihood) together with protein and UTR features using IsoformSwitchAnalyzeR [291] or tappAS [292], adding Biosurfer [379] when proteoform differences are central. When making protein‐level claims, prioritize candidates supported by Ribo‐seq and/or proteomics, and use structure‐oriented analyses selectively to vet the plausibility of disrupted folds or interfaces before committing to targeted functional experiments.

### Visualization

Long‐read transcriptome visualization should make full‐length isoform differences both easy to interpret and easy to verify, because many biologically important changes are subtle (junction shifts), end‐driven (alternative 5'/3' ends and poly(A) sites), or combinatorial, and are therefore missed by gene‐level plots (Figure 8F). The most useful views show complete isoform paths with relative or differential usage, splice junctions and exon connectivity with explicit read support, end and poly(A) heterogeneity, and locus‐level QC signals such as coverage dropouts, mapping artifacts, and agreement with matched short‐read RNA‐seq. When relevant, overlay functional annotations (ORFs, protein domains, NMD‐related features) so structural changes directly translate into testable hypotheses. A clear workflow is to present results in layers: gene‐level summary first, isoform models and usage next, and event/read‐support tracks last, while stating filtering thresholds to avoid over‐interpreting low‐confidence isoforms.

Tool choice naturally follows this layered goal. Genome browsers such as IGV [392] are indispensable for locus inspection and QC because they can display long‐read alignments, junction tracks, coverage, and short‐read support side by side, making it the most reliable option for validating candidates and diagnosing artifacts. For communicating isoform structure beyond raw alignments, Swan [393] provides graph‐based views that highlight isoform paths and end variability and generates compact gene‐level reports. If interactive exploration and annotation are needed, IsoVis [394] offers a web‐based isoform viewer and can map protein features onto exons to connect structure with predicted functional impact. For standardized, end‐to‐end reporting that links differential analysis outputs directly to figures, isoespy [395] is a practical choice. In routine use, combine IGV [392] for evidence checking with Swan [393] or IsoVis [394] for presentation, and add isoespy [395] when you need reproducible, pipeline‐aligned figure generation.

## CHALLENGES AND FRONTIERS

### Challenges: Library preparation, accuracy, throughput, and cost

The long‐read advantage of LRS enables characterization of transcript structures using full‐length molecular evidence rather than fragment‐based inference [396]. This improves the delineation of novel transcripts, splicing connectivity, transcript boundaries, fusion transcripts, and allele‐specific information [113]. However, scalable deployment in large cohorts and routine analysis workflows remains constrained by coupled bottlenecks. Incomplete transcript capture during library preparation limits the effective “full‐length” coverage actually achieved; low accuracy compromises base‐resolution fidelity in transcript structure characterization [20, 81]; low throughput limits detection of low‐abundance and rare transcription events; high cost restricts accessibility for single research groups and limits cohort size; and the lack of standardized analysis pipelines undermines result interpretability and cross‐batch comparability [397].

### Library preparation: Capturing the full‐length transcriptome

Library preparation remains a major bottleneck in realizing the full potential of long‐read transcriptomics. Achieving truly full‐length transcript capture, from the 5' cap to the poly(A) tail without internal dropout or fragmentation, is technically challenging. Current protocols face multiple interrelated limitations. First, reverse transcriptase processivity, despite improvements with engineered enzymes (e.g., TGIRT [398]), still declines on long and structured RNA templates, leading to truncated cDNAs that systematically underrepresent long transcripts and 5'‐terminal sequences. Second, template‐switching during RT can generate artifacts such as spurious polyadenylation sites [399]. Third, size‐selection steps, while helpful for enriching full‐length molecules, inevitably bias against very long transcripts (>10 kb). Fourth, for degraded specimens such as formalin‐fixed, paraffin‐embedded (FFPE) tissues, standard library preparation workflows often fail to produce qualified input, necessitating specialized protocols that are labor‐intensive and less scalable [400].

Progress in library preparation has been uneven. Cap‐trapping and poly(A) tail‐focused enrichment strategies improve end fidelity but do not directly address truncation or chimerism. Looking forward, further innovations are essential. These include engineered reverse transcriptases with enhanced strand displacement and thermostability; chemical treatments to reduce RNA secondary structure prior to RT; and microfluidic or droplet‐based protocols that minimize sample loss. Without such advances, even the most accurate LRS platforms will remain underutilized, as the input material itself fails to represent the full transcriptome completeness and complexity.

### Accuracy: Base‐level errors and transcript‐structure fidelity

LRS accuracy can be assessed across multiple linked layers: base‐level correctness, splice junction fidelity, exon‐chain reconstruction with well‐defined transcript boundaries, and consistent quantification across conditions. Together, these factors determine the stability of the inferred isoform repertoire and the robustness of downstream differential analyses [40, 41].

Among current LRS platforms, PacBio HiFi sequencing has established itself as the gold standard for accuracy. HiFi reads routinely achieve >99.9% consensus accuracy, delivering high per‐read correctness and structural concordance that support high‐confidence end‐to‐end transcriptome construction [18, 28]. In contrast, ONT and other emerging LRS platforms exhibit more variable error profiles that can compromise analytical precision. ONT error profiles are more context‐dependent, with prominent homopolymeric regions [118]. While ONT DRS retains native RNA characteristics, it is more susceptible to impacts from RNA stability and secondary structure, increasing ambiguity in transcript boundary assignment [38, 85, 401]. Beyond ONT, other emerging LRS platforms lack sufficient third‐party validation, and their accuracy profiles remain to be systematically evaluated against established benchmarks.

Accuracy is further shaped by library construction methods [402]. RT template switching and PCR amplification can introduce chimeras and artifactual transcripts that propagate into transcriptome reconstruction as spurious transcript models [62, 403]. Library designs that better constrain true transcript ends such as Cap‐trapping or poly(A) tail‐focused enrichment strategies can improve full‐length structure fidelity [88, 92, 93, 95, 96, 99, 100]. For ONT cDNA sequencing, approaches such as R2C2 can improve per‐read correctness by consensus construction, at the cost of effective depth and throughput [122].

Computational methods also play a critical role in accuracy improvement. Advances in basecalling algorithms have progressively reduced error rates for ONT platforms [13]. Basecaller updates can shift error modes and, in DRS, alter modification‐associated signals [53, 118, 126]. Downstream computational steps, including alignment filtering, splice junction correction, end processing, and transcript collapsing rules, directly influence the structural fidelity of full‐length transcripts. Key thresholds should therefore be aligned with study aims and made explicit to support cross‐study comparison [16, 41]. In addition, read allocation among highly similar transcripts and repeat‐adjacent loci remains intrinsically ambiguous; explicit assignment rules and uncertainty handling are essential for accurate quantification [40].

Looking forward, several avenues hold promise for further accuracy improvements. Continued refinement of nanopore chemistries and motor proteins may reduce raw error rates while preserving or increasing translocation speeds. Enhanced basecalling models trained on diverse RNA modification contexts could improve both sequence accuracy and epitranscriptomic signal resolution. The development of standardized reference materials and benchmarking frameworks would also benefit accuracy improvement.

### Throughput: Usable molecular evidence rather than nominal yield

Compared to NGS platforms such as Illumina NovaSeq, which routinely generate hundreds of Gb to a few terabases per run, some LRS platforms (e.g., PacBio Sequel II) face inherent throughput constraints that limit their scalability for large‐cohort studies. Given the relatively lower accuracy of LRS compared with NGS, throughput is better assessed by the amount of usable evidence than by nominal Gb yield alone. This is particularly critical for transcriptomics studies, where usable evidence depends on read quality, full‐length coverage, end completeness (especially at the 5' end), and effective per‐sample depth after demultiplexing. These constraints are amplified by the wide dynamic range of gene expression: highly abundant transcripts dominate sequencing capacity, whereas low‐abundance transcripts and rare splicing events require substantially more supporting molecules for reliable recovery [2].

Among LRS platforms, throughput varies considerably. PacBio platforms, while offering exceptional accuracy, are limited by the number of ZMWs per SMRT Cell. Each ZMW sequences only one molecule per run [40]. In traditional Iso‐Seq workflows, cDNA templates are substantially shorter than the polymerase's maximum synthesis capacity, leading to inefficient capacity utilization. The output of PacBio is shaped by library size distribution, loading efficiency, and the trade‐off between insert length and the number of passes per cDNA molecule [46].

ONT PromethION platform offers higher theoretical throughput, but output is primarily set by platform parallelization and run configuration. In ONT workflows, usable output depends on the fraction of active pores, chemistries, basecalling models, and template properties. Within the same ONT platform, DRS typically yields fewer reads and more pronounced 5'end coverage loss compared to cDNA sequencing [27, 85, 113, 404].

For emerging LRS platforms (e.g., those from QitanTech, CycloneSEQ), throughput claims (e.g., >100 Gb per run) remain to be independently validated. While these companies report impressive specifications, third‐party benchmarking studies are essential to assess whether real‐world performance aligns with advertised figures, particularly regarding read accuracy and length distributions, consistency across runs, and end‐to‐end coverage uniformity.

Several strategies have been developed to mitigate throughput constraints. Chemistry upgrades can increase reads per run across platforms. In PacBio, template‐organization strategies (e.g., Kinnex) can increase the information carried per read when typical transcript lengths are shorter than the platform's readable range [134]. When analyses target predefined genes or event classes, effective throughput can be increased by preferentially allocating sequencing capacity to molecules of interest. During library construction, targeted capture like TEQUILA‐seq increases the on‐target fraction under a fixed read budget [88]. At the stage of sequencing, adaptive sampling, implemented by ONT platforms, redistributes sequencing capacity in real time toward specified targets [405, 406].

Looking forward, further innovations in throughput enhancement are needed. For PacBio, increasing ZMW density and improving polymerase processivity could yield higher read counts per SMRT Cell. For ONT and other nanopore‐based platforms, developing more stable flow cell manufacturing processes would reduce inter‐run variability and improve throughput predictability. Continued advances in motor protein speed and processivity could increase translocation rates without compromising accuracy. Effective throughput is determined not only by platform specifications but also by library construction strategies, capacity allocation, and the alignment between study design and available evidence density. Ongoing innovations in template organization, targeted enrichment, and reusable nanopore architectures hold promise for the future.

### Cost: Declining but still a barrier for individual laboratories

Among the constraints facing LRS adoption, cost most directly limits cohort scale and routine adoption. For LRS, cost is better conceptualized as the total end‐to‐end investment required to produce qualified, usable data across the entire workflow, rather than the nominal price per run or per Gb [86]. This distinction matters because overall expenditure is typically dominated by cumulative attrition across multiple steps and by coverage requirements imposed by downstream tasks, rather than by any single reagent line item [407].

Hardware and consumables at the sequencing end constitute the most direct cost sources. The initial capital investment, maintenance, and auxiliary infrastructure required for mainstream LRS platforms elevate long‐term operational expenses, while single‐use core consumables (e.g., flow cells for ONT) represent substantial recurring expenditures [408]. Despite continuous technological iterations, LRS generally remains more expensive than NGS per unit of data. Estimated costs per Gb for CLR mode and HiFi mode generated by the PacBio Sequel II platform are approximately $13–$26 and $43–$86, respectively, whereas the ONT PromethION costs about $21–$42 [154], all of which exceed those of NGS platforms (<$10 per Gb) [409]. Notably, these figures may not reflect current pricing, as platform upgrades (e.g., PacBio Revio/Vega) and market dynamics have likely shifted costs downward. For emerging LRS platforms (e.g., QitanTech, CycloneSEQ), pricing remains largely opaque. While these companies may offer competitive per‐run or per‐Gb pricing, the lack of publicly available information and limited third‐party validation make it difficult to assess their true cost‐effectiveness. Additionally, direct comparisons based solely on per‐Gb metrics can be misleading because the value of LRS lies not in raw data volume but in the unique information provided by high‐accuracy full‐length reads.

Upfront sample preprocessing and library preparation are frequently underestimated contributors to total LRS cost. LRS‐based transcriptome characterization depends on high‐integrity RNA molecules. In degradation‐prone specimens such as FFPE tissues, standard automation often fails to yield qualified input, forcing more labor‐intensive extraction and QC workflows [400, 410]. Library preparation is also technically demanding, and degraded samples can incur high failure and repeat rates. Moreover, many applications (e.g., differential transcript expression) require sufficient molecule counts and replicates to separate true signals from technical noise, further increasing total investment.

Several practical strategies can reduce end‐to‐end costs. The first level is increasing usable yield per run, which amortizes fixed consumable costs across more data. Chemistry and workflow improvements, together with multiplexing, enable more efficient use of flow cells and reagents across samples [120, 411]. Higher‐throughput platforms such as PacBio Revio and ONT PromethION substantially reduce per‐sample costs when fully utilized. Since LRS operation is currently not fully robust, reducing failure and repeat rates through optimized protocols, automation and standardized experimental workflows represents another direction for cost reduction. At the study‐design level, targeted enrichment or selective sequencing can further reduce the cost per actionable evidence by concentrating reads on question‐relevant molecules [88]. Additionally, hybrid study designs that use long reads for transcript discovery and short reads for transcript quantification can substantially reduce the long‐read depth required to meet analytical objectives, thereby minimizing the consumption of high‐cost LRS resources and lowering the overall end‐to‐end cost [236, 412].

The cost trajectory for LRS is expected to continue downward as platform technologies mature. However, for individual laboratories, cost remains a substantial barrier. Although ONT offers portable, low‐entry‐barrier devices such as the MinION, the per‐sample or per‐Gb cost for small‐scale users is considerably higher than for large institutions that can fully utilize high‐throughput platforms like PromethION or Revio. This pricing structure creates a disparity where smaller research groups face disproportionately high per‐unit costs, limiting equitable access to LRS technologies. The economic viability of LRS, therefore, hinges on whether its higher sequencing costs are compensated by the added value of long reads. By providing end‐to‐end transcript structures, LRS can reduce ambiguity and false positives, thereby lowering the need for extensive downstream computation and follow‐up experiments.

### Challenges: Availability and performance of emerging LRS platforms

PacBio and ONT are commercialized worldwide with established distribution, reagent supply, and support networks, making them the most broadly accessible platforms across countries. In contrast, emerging nanopore sequencing platforms such as QitanTech are at present deployed primarily within China.

Unlike PacBio and ONT, most emerging LRS platforms lack large‐scale, publicly available datasets generated by third‐party studies, making it difficult to assess their real‐world performance on transcriptomics studies. The reagent ecosystem remains underdeveloped. For most of these platforms, there are no officially supported, specialized library preparation kits for full‐length cDNA or DRS, forcing users to adopt generic DNA workflows with suboptimal performance for transcriptomics. A substantial gap exists in upstream bioinformatics tools, particularly basecallers. Unlike ONT's well‐optimized and continuously updated Dorado pipeline, the basecalling and signal‐processing tools for emerging platforms are still nascent, with limited community support, lower accuracy, and less adaptability to transcriptomic data. Therefore, while these platforms hold promise for diversifying the long‐read landscape, their current limitations must be carefully considered. Rigorous, independent benchmarking by professional consortia will be highly valuable to establish objective performance metrics and guide their future application in transcriptomics studies.

### Challenges: Scalability, transcriptome complexity, and reference annotation

As LRS moves from single‐sample or small‐cohort studies toward population‐scale and clinical applications, scalability across large numbers of samples is becoming a central challenge [33, 343]. Multi‐sample transcriptome reconstruction requires explicit decisions about whether reads should first be pooled for joint isoform discovery or whether transcriptomes should be reconstructed per sample and subsequently merged. The recently proposed Join & Call and Call & Join framework formalizes these alternatives. Join & Call can increase sensitivity for rare or lowly expressed novel isoforms by pooling evidence across samples, whereas Call & Join is generally more scalable and may be preferable for highly replicated or large‐cohort studies when rare‐isoform discovery is not the primary objective [413]. Thus, future population and clinical LRS studies must balance discovery sensitivity, preservation of sample‐specific isoforms, computational cost, memory requirements, and downstream quantification consistency. These considerations also reinforce the need to report sample‐combination strategy, filtering criteria, merge thresholds, software versions, and transcript‐calling parameters as part of minimum workflow metadata.

A related challenge is the rapid expansion of detected transcript models. LRS routinely identifies large numbers of low‐abundance, tissue‐specific, condition‐specific, and incompletely characterized isoforms, raising important questions about how transcriptome complexity should be measured, filtered, interpreted, and incorporated into reference annotations. Recent work using perplexity, defined as the effective number of isoforms derived from Shannon entropy, provides a useful framework for quantifying isoform diversity without relying on arbitrary expression cutoffs [414]. Such approaches may help distinguish genes with many detected transcript models from genes with genuinely broad isoform usage.

At the annotation level, resources such as GENCODE, together with related efforts including MANE Select, GENCODE Primary, APPRIS, RefSeq harmonization, and community benchmarking initiatives, provide essential frameworks for balancing transcriptome completeness with usability, stability, and clinical interpretability [40, 415, 416, 417]. However, the growing number of LRS‐supported isoforms also creates a tension between comprehensive annotation and practical usability. The field must avoid treating every detected transcript as equally interpretable, while also ensuring that genuine tissue‐, development‐, or disease‐relevant isoforms are not removed by overly aggressive filtering.

These issues are particularly important for de novo genome annotation, especially in non‐model organisms [418, 419]. In such settings, long reads can provide full‐length evidence for gene models, UTRs, splice isoforms, lncRNAs, TE‐derived transcripts, and species‐specific isoforms, but annotation transfer and manual curation are often limited [420]. LRS‐based annotation should therefore be combined with standardized structural classification, orthogonal evidence from short reads, proteomics, conservation or synteny, and transparent confidence labels [25, 40, 421]. Such integrated strategies will be essential for producing transcript annotations that are both comprehensive and biologically interpretable.

### Challenges: Standardization of bioinformatics methods

LRS technology has fundamentally reshaped the dimensions of exploring transcriptomic complexity, including alternative isoform regulation [343], ranging from single‐cell isoform resolution [73, 422], and spatial transcriptomics [423, 424] to direct RNA modification detection [27, 113] and proteogenomic integration [425, 426]. However, the rapid emergence of specialized methods and computational tools has also introduced a major field‐wide limitation: standardized workflows and benchmarking frameworks are still lacking for most steps of LRS experimentation and analysis. This “toolbox explosion”, while reflecting strong innovation, is increasingly a bottleneck for methodological maturation and clinical translation (Table S3).

Although various methods share the common goal of resolving full‐length transcript isoforms and their regulatory characteristics, LRS workflows exhibit significant heterogeneity at almost every step. At the library construction level, protocol differences between platforms such as ONT and PacBio are pronounced [427]. Even within the same platform, specific construction strategies are required for different RNA biotypes (e.g., tRNA [428], circRNA [429], and various non‐coding RNAs (ncRNAs) [430]). These methodological differences introduce protocol‐specific biases, complicating cross‐study comparisons and meta‐analyses [431].

Heterogeneity is even more pronounced at the computational level. For transcript identification and quantification alone, numerous tools (e.g., IsoQuant [214], Bambu [220], StringTie3 [213], FLAIR [212], Isosceles [216], and miniQuant [141]) implement different approaches to read alignment, splice‐junction inference, and abundance estimation. Benchmarking studies show significant differences in sensitivity, precision, and false positive rates among these tools, particularly for complex genes and low‐abundance isoforms [40, 432, 433]. Similarly, in the field of RNA modification detection based on nanopore DRS data, a large number of methods based on statistics, machine learning, or integrated into basecalling pipelines have emerged, such as Nanocompore [434], xPore [435], TandemMod [150], CHEUI [436], and Dorado's (https://github.com/nanoporetech/dorado) modification detection models, which rely on different assumptions, training data, and signal processing strategies, often leading to discrepant results [437, 438, 439]. This fragmentation also exists in poly(A) tail length estimation: tools such as tailfindr [311], and Dorado (https://github.com/nanoporetech/dorado/) employ different signal segmentation and normalization methods, resulting in variations in tail length distributions. For APA analysis, methods are also diverse, ranging from isoform inference‐based approaches [214, 220] to dedicated peak identification pipelines such as APALORD [306] and LAPA [307], with no current consensus on how to optimally identify and quantify poly(A) site usage.

While the current “tool explosion” has driven technological innovation, it has also introduced several key obstacles. First, irreproducibility becomes a prominent issue: choices of different tools and parameters can lead to conflicting biological conclusions, severely undermining the reliability of discoveries based on LRS [40, 41, 437, 439]. Second, the difficulty of method selection is increasingly apparent: the lack of standardized benchmarks presents a significant challenge for researchers, especially newcomers to the field, in selecting appropriate tools for specific biological questions [40, 44]. Third, hindering clinical translation is a realistic bottleneck: if LRS is to realize its potential in precision medicine [86, 440], regulatory agencies and clinical laboratories require validated, standardized workflows with clear performance metrics. Finally, missed integration opportunities are regrettable: fragmentation between analytical layers (isoform identification, modification detection, poly(A) analysis) prevents us from fully leveraging the potential of LRS data. For example, integrating information on isoforms, modifications, and poly(A) tails could reveal more complex gene regulatory mechanisms [441, 442].

Addressing these challenges requires a coordinated community effort to establish a multi‐dimensional algorithm standardization framework. First, build benchmark datasets and ground truth resources. Comprehensive reference datasets with known ground truth are the cornerstone of algorithm standardization. Consortia like LRGASP have made significant progress in benchmarking isoform identification and quantification methods across platforms and analysis pipelines [40]. Future expansion is needed, including synthetic spike‐in controls (with defined isoform structures, modification statuses, and poly(A) tail lengths) [443, 444, 445], simulation‐based benchmarks (allowing systematic evaluation under controlled conditions) [446, 447], and cross‐platform reference samples (sequencing the same biological material with multiple technologies) [432]. These resources should be designed to reflect the full complexity of native transcriptomes.

Second, establish standardized quality metrics and reporting guidelines. A core reason for the difficulty in comparing tools is the inconsistency in evaluation metrics. The community should reach a consensus on a set of standardized performance metrics for each analytical task: for isoform quantification, unify precision, recall, F1‐score, and correlation with ground truth abundance [40, 432]. For RNA modification detection, specify area under the ROC curve, precision‐recall curves, False Discovery Rate, and accuracy of stoichiometric estimation [437, 439, 440]. For poly(A) tail analysis, define mean absolute error, correlation with orthogonal measurements, and inter‐replicate variability [443]. For APA analysis, unify criteria for identification accuracy and quantitative consistency [303]. In parallel, minimum reporting standards for LRS studies should require transparent disclosure of library protocols, sequencing settings, basecalling models, software versions, and parameter choices [40].

Third, organize community‐endorsed benchmarking challenges. Learning from successful models in other fields (e.g., CAGI for variant effect prediction [448]), the long‐read community should regularly organize benchmarking challenges focused on specific analytical tasks. Recent efforts like the “RMaP Challenge” [449] for RNA modification detection are a positive step forward. These challenges should use blinded datasets where ground truth is hidden, attracting diverse research teams to apply their methods, providing systematic comparisons, and fostering iterative algorithm improvement.

Fourth, develop modular, interoperable workflow frameworks. Standardization does not mean mandating a single “best” tool for every task, but rather promoting the development of modular, interoperable workflows that allow users to combine validated components while maintaining reproducibility. Platforms like Nextflow [450], combined with containerization technologies, make it possible to create portable analysis pipelines encapsulating best practices. For example, the MasterOfPores pipeline [451] provides a modular and reproducible workflow specifically designed for Oxford Nanopore DRS data, integrating tools for basecalling, alignment, and modification detection.

Fifth, enhance machine learning model cards and training data transparency. As deep learning becomes increasingly central to LRS data analysis, from basecalling [161] to modification detection [150, 452, 453] and other key tasks like isoform quantification [216], establishing standards for model documentation and transparency is crucial. Adopting frameworks like “model cards” would require developers to disclose key information such as training data sources, model architecture, intended use, and known limitations [454].

Sixth, proactively address integration with emerging AI and foundation models. The rapid development of large language models and genomic foundation models presents both opportunities and challenges for algorithm standardization [455]. Standardization efforts must continuously evolve to address new issues such as comparing foundation models with traditional methods, adapting pre‐trained models, and model interpretability.

The key to LRS technology maturing from a research tool to a platform for biological discovery and clinical application lies in establishing robust, community‐validated standardization frameworks. This does not imply a rigid “one‐size‐fits‐all” model, as different biological questions will continue to require tailored analytical strategies. Instead, standardization should provide shared benchmarks for fair comparison, transparent reporting to ensure reproducibility, modular workflows to facilitate the adoption of best practices, and community governance to guide methodological evolution. By addressing current fragmentation and establishing these foundational standards, the field can accelerate the translation of LRS technologies into reliable tools for deciphering the complexity of RNA biology, ultimately realizing their potential as cornerstones of precision medicine and multi‐omics integration [44, 86, 440].

These standardization efforts are not abstract goals. They are urgently needed to support the responsible development of the very technologies that are redefining the landscape of transcriptomics. The following section introduces the latest frontiers of LRS: single‐cell resolution, spatial mapping, direct RNA modification detection, and multi‐omics integration (Figure 1C). Each of these areas exemplifies both the remarkable potential and the analytical fragmentation that community‐driven standardization seeks to address.

### Frontiers: Long‐read single‐cell transcriptomics

Long‐read single‐cell RNA‐seq (scRNA‐seq) represents a cutting‐edge approach. By sequencing full‐length cDNA, this technology overcomes the limitations of existing NGS‐based scRNA‐seq techniques, which typically capture only the 3' or 5' ends of transcripts [456]. LRS platforms (e.g., ONT) have been successfully integrated into droplet‐based scRNA‐seq systems (e.g., 10× Genomics) [73]. This integration has given rise to a series of specialized long‐read scRNA‐seq methods, including ScISOr‐Seq [457], CELLO‐seq [273], ScNaUmi‐seq [458], scCOLOR‐seq [459], LR‐Split‐seq [460], scTaILoR‐seq [461], and FlsnRNA‐seq [462] (Figure 9A). This technological breakthrough enables the study of complete RNA structural variants at single‐cell resolution.

**FIGURE 9:**
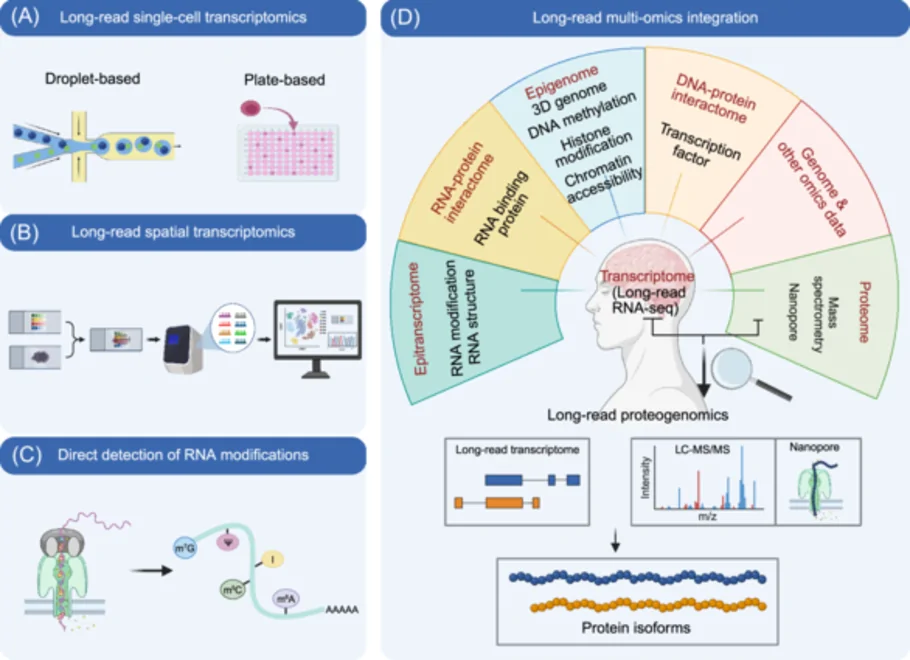
Emerging frontiers for long‐read transcriptomics studies. (A) Long‐read single‐cell transcriptomics integrates LRS with single‐cell resolution, enabling isoform‐level characterization of cellular heterogeneity. Key technical routes include droplet‐based high‐throughput methods and plate‐based approaches for deep coverage, both leveraging cell barcodes and UMIs for accurate molecule assignment. (B) Long‐read spatial transcriptomics preserves spatial coordinates while capturing full‐length isoform information, enabling the study of spatially‐resolved alternative splicing and polyadenylation within tissue sections. (C) Direct detection of RNA modifications by direct RNA sequencing preserves native RNA molecules and allows simultaneous profiling of RNA sequence, multiple modification types, and higher‐order structural features at single‐molecule resolution, bypassing antibody enrichment or chemical conversion. (D) Long‐read multi‐omics integration enables concurrent or coordinated profiling of multiple molecular layers from the same sample or even the same molecule, including genome, epigenome, transcriptome and epitranscriptome, and proteome, providing a unified framework from genotype to phenotype with full isoform resolution. LC‐MS/MS, liquid chromatography‐tandem mass spectrometry; UMI, unique molecular identifier. Created in BioRender. Li, J. (2026) https://BioRender.com/8sdfzpb.

While traditional NGS‐based scRNA‐seq has significantly advanced our understanding of cellular heterogeneity, the reliance on short reads imposes inherent limitations. Due to the limited read lengths (50–300 bp [456]), these methods can only quantify gene expression by capturing tags at the ends of transcripts, losing most of the intermediate sequence information [456]. Consequently, NGS‐based scRNA‐seq cannot effectively resolve complex transcription events (e.g., alternative splicing, gene fusion). With the application of LRS, single‐cell transcriptome analysis has undergone a dimensional leap from the gene‐level to the isoform‐level [73]. Full‐length transcript sequencing enables identification of cell type‐specific alternative splicing events, APA sites, and fusion genes, revealing the transcriptomic complexity masked by NGS‐based scRNA‐seq studies.

Long‐read scRNA‐seq has two main technical routes (Figure 9A): droplet‐based high‐throughput methods exemplified by 10× Genomics, which are suitable for profiling complex cell populations, and plate‐based approaches (e.g., SCAN‐seq and SCAN‐seq. 2), which provide higher transcript coverage depth and detection sensitivity. The latter is particularly suited for studying scenarios (e.g., mammalian early embryonic development) with limited cell numbers requiring deep coverage [423, 463]. Recent advances have further expanded full‐length single‐cell transcriptomics toward total RNA analysis, enabling comprehensive capture of both polyadenylated and non‐polyadenylated transcripts [431].

A defining feature of scRNA‐seq protocols is the use of cell barcodes and UMIs to assign reads to individual cells and molecules. In the context of LRS, accurate recovery of these sequences presents distinct challenges. Cell barcode recovery is relatively robust, as each barcode is supported by many transcripts; in practice, barcode assignment can be further aided by matched short‐read data when needed. In contrast, UMI recovery is substantially more challenging. Because each UMI typically corresponds to a small number of transcripts, sequencing errors in long reads can introduce “scattering” effects, where reads derived from the same molecule are erroneously split into multiple UMI groups. This leads to sequence‐ and expression‐dependent biases in transcript quantification. Early approaches such as ScNaUmi‐seq (Sicelore) [458] addressed this issue by leveraging matched short‐read sequencing to recover UMI information; however, this strategy can be cost‐prohibitive, as sufficiently deep short‐read sequencing is required to reconstruct UMI whitelists. More recent methods, such as Longcell [464] and UMI‐nea [465], resolve these biases through improved UMI recovery and clustering directly from long‐read data.

Beyond their canonical role in molecule counting, UMIs provide additional value in long‐read scRNA‐seq. When properly recovered, they can support error correction and facilitate transcript disambiguation, as discussed in the “Error Correction” section. Indeed, advances in UMI‐aware clustering algorithms have largely alleviated earlier limitations associated with high error rates in LRS.

Beyond the pre‐processing step, a series of specialized pipelines has been developed. FLAMES [121] integrates core modules such as cell barcode/UMI assignment, transcript identification and quantification, alternative splicing analysis, and mutation detection. The latest version of Bambu [220] supports context‐aware transcript identification and quantification for long‐read scRNA‐seq data, and Isosceles [216] is compatible with single‐cell, pseudo‐bulk, and bulk data analysis. Additional long‐read scRNA‐seq analysis tools have been developed: for example, scNanoGPS [466], a comprehensive toolbox, enables characterization of genotype and phenotype in single cells without requiring NGS data or predefined barcode whitelists, Isopod [467] can detect differential transcript usage, JAFFAL [331] supports fusion gene detection, and ScisorWiz [468] provides visualization modules for differentially expressed transcripts.

Recent applications of long‐read scRNA‐seq have yielded novel biological insights across multiple frontiers. It was employed to reconstruct the transcriptomic dynamics during endothelial‐to‐hematopoietic transition in mouse embryonic development, revealing the fine regulatory control at the isoform level during this critical developmental event [469]. In ovarian cancer, by long‐read scRNA‐seq data, the fusion between *IGF2BP2* and *TESPA1* genes was identified, and the mechanism of mesothelial cell transformation into cancer‐associated fibroblasts was uncovered [470]. ScNaUmi‐seq was applied to analyze embryonic mouse brains, not only defining cell‐type‐specific splicing patterns but also achieving precise detection of RNA editing events at single‐cell resolution [458]. Long‐read scRNA‐seq was performed on mouse retina, demonstrating that isoform‐level information is crucial for refining cell classification [471]. The significance of these studies is that single‐cell LRS is not merely a “patch” to existing cell atlases. Instead, by introducing the new resolution dimension of isoforms, it is rewriting our understanding of cellular heterogeneity, developmental regulation, and disease mechanisms, providing a critical bridge connecting genotype and phenotypic complexity in the era of precision medicine.

Importantly, long‐read scRNA‐seq not only refines cell type definitions but also enables the study of isoform switching within individual cells. Early studies suggested substantial isoform uniformity within single cells, but interpretation was complicated by low coverage and dropout effects. The challenges posed by sparse sampling were further explored by Buen et al. [472], who warned against the spurious appearance of binary splicing created by dropout events. After accounting for these technical sources of confounding, Fu et al. [464] systematically quantified isoform usage and demonstrated that both intra‐cellular isoform diversity and inter‐cellular isoform heterogeneity coexist, establishing isoform switching as a genuine and biologically meaningful feature of single‐cell transcriptomes.

### Frontiers: Long‐read spatial transcriptomics

The function of tissues and organs is determined not only by the identity and abundance of molecular species but also by their precise spatial organization. Traditional single‐cell sequencing methods dissociate tissues, thereby losing the native spatial context that is critical for understanding cell‐cell interactions, tissue architecture, and regional specialization. Spatial omics technologies address this limitation by mapping molecular features (e.g., chromatin, transcripts, proteins, metabolites) directly onto tissue sections, preserving spatial coordinates while enabling high‐dimensional molecular profiling [473, 474, 475].

The spatial omics landscape encompasses diverse modalities and resolutions [473, 476]. At the protein level, imaging‐based methods such as multiplexed immunofluorescence (e.g., CODEX [477]) enable simultaneous detection of dozens of protein markers. At the RNA level, there are two approaches: imaging‐based methods (e.g., MERFISH [478], seqFISH+ [479]) that visualize individual RNA molecules in situ through combinatorial labeling and sequential imaging; and sequencing‐based methods (e.g., 10× Genomics Visium [480], Slide‐seq [481], Stereo‐seq [482]). More recently, multi‐omic spatial technologies have begun to integrate genomic, transcriptomic, proteomic, and epigenomic measurements from the same tissue section, enabling holistic characterization of cellular states within their native microenvironment [475].

Traditional spatial transcriptomics technologies (e.g., 10× Genomics Visium platform, Slide‐seq) primarily rely on NGS. These methods label RNA molecules with spatially barcoded tags, enabling high‐throughput profiling of gene expression at defined spatial coordinates. However, due to the limited read lengths of NGS, these technologies fail to preserve full‐length transcript information. Given that approximately 95% of multi‐exon genes in the human genome undergo alternative splicing [483], this limitation has significant consequences: while one can quantify the overall expression level of a gene at a specific spatial spot, it is difficult to determine which specific isoforms of that gene are expressed at that location [425, 484]. As a result, traditional spatial transcriptomics cannot resolve the transcriptome complexity in situ, thereby hindering efforts to link spatial expression heterogeneity directly to functional regulation at the isoform level [424, 425].

LRS introduces a new dimension to spatial transcriptomics (Figure 9B) by enabling full‐length transcript information in situ [475]. DRS can further preserve base modification information on the full‐length RNA molecules, opening the door to long‐read spatial transcriptomics and epitranscriptomics [424, 425]. Methodological advances have rapidly expanded the resolution and throughput of long‐read spatial transcriptomics. Several techniques have emerged. For instance, Spl‐ISO‐Seq achieves near‐single‐cell resolution (10 μm) while increasing read lengths two‐ to threefold through exon enrichment and long cDNA selection; and its successor, Spl‐ISO‐Seq2, pushes resolution to 500 nm, reaching sub‐cellular level [425, 485]. In parallel, Fullscope‐seq uses programmed cDNA concatenation to enable full‐length single‐molecule spatial transcriptomics across a large field of view at single‐cell resolution and is compatible with multiple long‐read sequencing platforms [486]. On the computational front, dedicated tools have emerged: Spl‐IsoQuant‐2 [487] for barcode calling and isoform assignment from spatially barcoded long reads, Spl‐IsoFind for detecting cell‐type‐specific spatially variable isoforms [487], and Longcell [464] for recovering barcodes, correcting errors, and modeling splicing diversity, with systematic benchmarks now providing practical guidance for method selection [44].

However, a fundamental challenge remains for conventional spot‐based spatial barcoding technologies: each spot may contain transcripts from multiple cells and cell types. Consequently, while long‐read spatial transcriptomics can detect spatial isoform switching, it is difficult to attribute these events to specific cell types, as observed signals reflect aggregated contributions from heterogeneous populations. Recently, SpotGLM [488] introduced a mixture‐aware framework for cell type‐resolved isoform‐level inference from spatial data. By modeling spot‐level isoform proportions as mixtures of cell type‐specific signals, it enables direct testing of cell type‐specific, spatial context‐dependent isoform switching. Frontier research has begun to leverage these technologies to uncover critical biological principles. For example, Spl‐ISO‐Seq was used to analyze human visual cortex samples from pre‐adolescent (8–11 years) and adolescent (16–19 years) individuals [425]. This study revealed that the regulatory intensity of alternative splicing and polyadenylation was higher in the cerebral cortex than in white matter, whereas the opposite was observed for oligodendrocytes [424]. The SiT method [423], and later, SpotGLM [488], was used to reveal regional isoform switching between different layers of the mouse olfactory bulb. More recently, Spl‐ISO‐Seq2 identified cell‐type‐specific spatially variable isoforms across multiple mouse brain cell types, including spatial isoform patterns that could not be explained solely by differences in cell‐type composition [487]. In the primate brain, Fullscope‐seq revealed extensive differential transcript usage and major isoform switching across cortical layers, cell types, and brain regions, with many of these events enriched in genes associated with neuropsychiatric disorders [486]. Collectively, these studies demonstrate that long‐read spatial transcriptomics can anchor dynamic transcriptional events to specific anatomical locations and cell types. This capability offers an unprecedented panoramic view for understanding the functional compartmentalization of complex tissues, particularly the brain, as well as the spatial regulation of development and disease progression.

### Frontiers: Direct detection of RNA modifications

A companion review provides a comprehensive assessment of DRS‐based RNA modification detection methods, benchmarking strategies, and remaining challenges [21]. Therefore, we do not reiterate those technical details in this review.

Beyond the canonical four bases, RNA molecules carry a diverse array of chemical modifications, with over 170 types currently known, widely distributed across nearly all RNA species [489, 490]. These modifications constitute a critical layer of gene expression regulation by regulating transcription, RNA splicing, export, stability, and translation [491, 492]. Advances in high‐throughput detection technologies, coupled with the identification of writers (e.g., methyltransferases), erasers (e.g., demethylases), and readers (binding proteins), have greatly expanded our understanding of how RNA modifications contribute to normal development and disease pathogenesis [491, 492]. Among the diverse modification landscape, m^6^A is the most abundant internal mRNA modification and has emerged as a paradigm for post‐transcriptional regulation [493]. Beyond m^6^A, a growing number of non‐m^6^A modifications, such as inosine (i.e., A‐to‐I editing), pseudouridine (Ψ), 5‐methylcytosine (m^5^C), N^7^‐methylguanosine (m^7^G), N^1^‐methyladenosine (m^1^A), and 2'‐O‐methylated nucleotides (Nm), are increasingly recognized for their essential roles in RNA metabolism [491].

Liquid chromatography‐tandem mass spectrometry enables unbiased quantification of total RNA modification abundance in a sample but lacks information on modification locations or sequence contexts. Over the past two decades, a diverse array of NGS‐based approaches for transcriptome‐wide RNA modification mapping has been developed, each with distinct strengths and limitations across three dimensions: input material requirements, base resolution, and quantitative accuracy. These methods typically rely on specific antibodies that capture modified RNAs, or chemical/enzymatic treatments that convert modification signals into detectable perturbations during RT (e.g., mutation signatures, truncation events) [491, 494].

For m^6^A, antibody‐based immunoprecipitation methods (e.g., MeRIP‐seq [495], m^6^A‐seq [496]) are widely used but have resolutions (50–300 bp) constrained by the enrichment characteristics of antibodies [495, 496]. Subsequent crosslinking‐based methods (e.g., miCLIP [497]) improve resolution to near‐nucleotide level by exploiting UV‐induced crosslinking between antibodies and RNA, though they require large input amounts. Antibody‐free approaches (e.g., MAZTER‐seq [498] and m^6^A‐REF‐seq [499]) achieve single‐base resolution through enzymatic cleavage but are constrained by dependence on specific sequence motifs. More recently, quantitative base‐resolution methods (e.g., m^6^A‐SAC‐seq [500], GLORI [501], and eTAM‐seq [502]) have enabled accurate stoichiometry measurement by converting either methylated or unmethylated adenosines.

For other modifications, analogous strategies have been developed. Ψ mapping relied on CMC chemistry that induces RT truncation (e.g., Ψ‐seq [503]), or base transitions (e.g., BID‐seq [504]) that enable quantification. m^5^C detection has historically employed bisulfite sequencing, which converts unmethylated cytosines to uracil but causes severe RNA degradation [505].

Despite their widespread use, NGS‐based methods share common conceptual limitations. First, they rely on indirect readouts rather than direct measurement of the modification itself. Second, the requirement for RNA fragmentation and NGS precludes the study of long‐range modification coordination, such as whether multiple modification sites on the same transcript co‐occur or are mutually exclusive. Third, each modification type requires a bespoke protocol, making simultaneous profiling of multiple modifications on the same molecule technically challenging.

DRS represents a fundamental departure from indirect detection paradigms. The core concept of direct RNA modification detection technologies is to utilize nanopore sequencing platforms, particularly ONT's DRS system, to enable complete RNA molecules to pass through a nanopore protein (Figure 9C). Different nucleotides generate characteristic ionic current signal perturbations as they traverse the nanopore. By analyzing these signals, both the sequence information and modification status of the RNA can be simultaneously obtained [27, 113, 434]. The advantage of this technical approach lies in its ability to resolve the modification map of full‐length transcripts at the single‐molecule level without relying on antibody enrichment or chemical conversion, thereby opening a new technological dimension for epitranscriptomics research [38, 81]. This technical architecture confers several transformative advantages [27, 149]. First, DRS achieves “non‐destructive” modification detection. RNA molecules are sequenced in their native state, preserving all endogenous modifications and avoiding PCR‐introduced biases and artifacts [113]. Second, the single‐molecule resolution enables researchers to resolve the “phase” relationships of modifications within transcripts [150]. Third, DRS allows mapping of modifications on full‐length transcripts. For example, it enables the identification of known modification sites on full‐length 16S rRNA [84], and facilitates stage‐resolved epitranscriptomic analysis of rRNA maturation in archaea [506].

In recent years, multiple studies have demonstrated the transformative impact of DRS on RNA modification detection [38]. For example, using ribosomal RNAs from *E. coli* and *S. cerevisiae* as model systems, studies systematically validated that DRS could simultaneously detect endogenous modifications (*e.g*., Ψ, Nm, m^5^C) and exogenously introduced chemical probes [114]. On this basis, some methods (*e.g*., PORE‐cupine [507], nanoSHAPE [114]) have been developed for single‐molecule probing of RNA higher‐order structure.

Algorithmic innovations have further expanded the field. Early DRS‐based RNA modification detection tools (e.g., EpiNano for m^6^A [147]) rely on post‐basecalling error patterns, while subsequent approaches (e.g., m^6^Anet for m^6^A [508], NanoPsu for Ψ [509], CHEUI for m^5^C [436], DeepEdit for Inosine [510]) leverage raw current signals with machine learning models. More recently, the deep learning framework ORCA was published [441]. ORCA leverages DRS with domain adversarial learning and transfer learning to simultaneously detect and quantify diverse RNA modification types. In parallel, the performance of different basecalling models for RNA modification detection was systematically evaluated, demonstrating that high‐accuracy models substantially improve detection accuracy [511], while a computational framework for de novo basecalling at single‐molecule, single‐base resolution was developed, enabling parallel detection of multiple modification types [159].

Despite these advances, several challenges persist [512]. First, DRS raw read accuracy remains lower than that of PacBio HiFi and ONT cDNA sequencing, with basecalling errors that can confound modification detection, particularly for modifications with subtle current signatures. Second, the field lacks gold standards including ground‐truth RNA molecules with specific modifications at given positions and reference datasets. Third, computational methods for modification calling vary widely in sensitivity, specificity and runtime, and there is no systematic benchmarking framework to guide users in selecting appropriate tools for their study context.

In addition to DRS, alternative LRS strategies (*e.g*., DART‐seq [513]) have also been reported. DART‐seq couples APOBEC1 deaminase with YTH‐domain fusion proteins to convert m^6^A sites into C‐to‐T mutations during RT. DART‐seq can be combined with LRS (*e.g*., PacBio or ONT cDNA sequencing), offering an orthogonal approach to achieve full‐length, isoform‐resolved m^6^A profiling without requiring DRS. This highlights that mapping modifications on the same full‐length RNA molecules can be pursued through multiple technological avenues.

Collectively, DRS has propelled RNA modification research from the traditional paradigm of “single modification, average signal” to a new phase of “co‐detection of multiple modifications, single‐molecule configuration analysis”, providing critical technical support for decoding the complex regulatory networks of the epitranscriptome.

### Frontiers: Long‐read multi‐omics integration

The advent of LRS has enabled a paradigm shift from isolated analysis of individual molecular layers toward integrated multi‐omic profiling on the same samples (e.g., DNA, RNA, protein). The value of long‐read transcriptome sequencing extends beyond single‐dimension resolution to its unique role as a hub for multi‐omics integration (Figure 9D). In addition to long‐read transcriptomics methods, a range of LRS‐based omics technologies have been developed over the years [13, 14, 18].

At the genome level, LRS enables telomere‐to‐telomere (T2T) genome assemblies, resolving previously inaccessible repetitive regions and structural variants [514]. Beyond sequence determination, PacBio and ONT platforms can directly detect epigenomic marks without additional chemical treatments. For example, ONT's raw electrical signals are sensitive to DNA methylation (e.g., 5mC), enabling direct detection of endogenous modifications during sequencing. To extend this capability to other epigenomic features (e.g., chromatin accessibility, nucleosome positioning, histone modifications), a suite of long‐read assays has been developed.

Multi‐epigenomic approaches (*e.g*., MeSMLR‐seq [515], SMAC‐seq [516], nanoNOMe [517], Fiber‐seq [518]) employ exogenous methyltransferases (e.g., GpC‐specific M. CviPI for 5mC labeling, context‐free Hia5 for m^6^A labeling) to label open or nucleosome‐free regions. This enables simultaneous detection of chromatin accessibility, nucleosome occupancy, and endogenous DNA methylation (e.g., 5mC at CpG sites) on single DNA molecules. For histone modification and protein‐DNA interaction mapping, techniques such as DiMeLo‐seq [519], nanoHiMe‐seq [520], and BIND&MODIFY [521] construct fusion proteins combining protein A with methyltransferases (e.g., Hia5), enabling targeted deposition of exogenous m^6^A at antibody‐bound genomic regions. These methods reveal histone modification profiles and transcription factor binding sites alongside endogenous 5mC. For 3D genome architecture, methods such as Pore‐C [522] and HiPore‐C [523] apply proximity ligation to cross‐linked chromatin, followed by LRS to capture multi‐way chromatin interactions on single DNA molecules.

At the transcriptome and epitranscriptome levels, DRS provides a solution for simultaneous detection of RNA sequence, modifications, and higher‐order structural features. Beyond base modifications, innovative assays (e.g., PORE‐cupine [507], nanoSHAPE [114], Nano‐DMS‐Map [524]) have extended DRS to probe RNA secondary structure through chemical probing strategies. For mapping RNA‐protein interactions, RNA base editor‐based strategies (e.g., MAPIT‐seq [525]) fuse RNA‐binding proteins with adenosine deaminases (ADAR) or cytidine deaminases (e.g., APOBEC), introducing mutations (A‐to‐I or C‐to‐U) at or near binding sites. Using similar RNA base editor principles, LRS‐based translatome mapping approaches (e.g., LR‐Ribo‐STAMP [526]) have also been developed.

At the proteome level, emerging studies have shown that nanopores can directly sequence proteins [527, 528, 529]. As proof‐of‐concept studies, Yu et al. presented an enzyme‐free method for unidirectional, slow transport of full‐length proteins through nanopores, achieved by combining a chemically resistant α‐hemolysin nanopore with a high‐concentration guanidinium chloride buffer that drives protein translocation via electroosmotic flow [527]. Similarly, Sauciuc et al. demonstrated that introducing sets of charges spaced ~1 nm apart within the CytK nanopore lumen generates an electroosmotic flow capable of driving unidirectional transport of unstructured natural polypeptides against a strong electrophoretic force [528].

The power of integrated long‐read multi‐omics has been demonstrated in several landmark studies. The T2T Consortium's 2022 *Science* special issue exemplified the potential of combining long‐read genome, epigenome, and transcriptome [530]. Hoyt et al. generated complete genomic and epigenetic maps of human centromeric repeats, integrating ONT‐based DNA methylation profiling with transcriptomic analysis to reveal the transcriptional and epigenetic landscape of previously inaccessible repetitive elements [531]. In another study, long‐read genome sequencing and DRS were applied to yeast to simultaneously decode complete genome structure, DNA modifications, transcription, and RNA modifications, thereby revealing how genetic and epigenetic features translate into transcripts [532].

Extending beyond RNA‐centric views, long‐read proteogenomics bridges RNA isoforms to protein products. Traditional proteomics often fails to capture sample‐specific or rare isoforms due to limitations in reference databases. Long‐read RNA‐seq addresses this gap by enabling the construction of sample‐specific protein sequence databases that capture full‐length isoform diversity. This paradigm was established by integrating PacBio and ONT RNA‐seq with mass spectrometry, identifying hundreds of protein isoforms absent from standard databases [425]. Applying this approach to cancer research, long‐read RNA‐seq was combined with multidimensional proteomics in Ewing sarcoma cells, detecting over 40,000 isoforms and identifying thousands of putative non‐canonical proteoforms, thereby exposing hidden immunopeptidome and neoantigen targets [426]. Cross‐platform integration further enhances characterization: for instance, PacBio Iso‐Seq and ONT DRS were combined in *Arabidopsis* to improve proteoform identification from complex splicing patterns [533]. Collectively, these studies propel the field from gene‐centric inference toward isoform‐resolved, sample‐specific proteome characterization.

The next frontier lies in developing truly unified workflows that capture multiple omics layers simultaneously from the same sample or even the same molecule. Emerging technologies are beginning to realize this vision. For instance, the combination of ONT‐based genome sequencing, DRS, and proteome sequencing from the same biological sample could enable the comprehensive characterization of the central dogma, from genotype to transcriptome to proteome, with full isoform resolution.

## CONCLUSION

LRS is shifting transcriptomics from gene‐level quantification to isoform‐resolved interpretation by enabling direct measurement of exon connectivity, transcript boundaries, and transcription in repetitive regions, including transposable elements. This review provides an end‐to‐end, implementation‐oriented roadmap that connects experimental design choices, platform selection, library preparation, enrichment or targeting strategies, and throughput‐accuracy trade‐offs to the downstream computational steps needed to convert raw data into curated transcript models and reliable quantification. We also summarize how improvements in basecalling, quality control, alignment, error correction, transcript identification and quantification, and TE‐aware analyses jointly determine isoform‐catalog accuracy and the robustness of biological conclusions.

Long reads further enable integrated analysis of key layers of transcriptome complexity, including alternative splicing, ATSS, APA, poly(A) tail profiling, circular RNAs, fusion transcripts, and ASE, within a unified isoform‐centric framework. This foundation supports downstream studies such as differential transcript expression, differential splicing, isoform switching, functional consequence annotation, and isoform‐level regulatory network inference. Despite rapid progress, practical adoption is still constrained by cost, limited effective depth for low‐abundance events, platform‐ and protocol‐specific biases, and inconsistent benchmarking and standardization. Continued advances in sequencing chemistry, signal modeling, reference materials, and reproducible modular workflows will be critical for making long‐read transcriptomics more routine, comparable across studies, and suitable for clinical translation.

## AUTHOR CONTRIBUTIONS

**Dingjie Wang**: Conceptualization; supervision; funding acquisition; visualization, project administration; resources; writing—original draft; writing—review and editing. **Yunhao Wang**: Conceptualization; supervision; funding acquisition; visualization; project administration, resources, writing—original draft, writing—review and editing. **Chuwen Xu**: Writing—review and editing; visualization, writing—original draft; data curation. **Jia Li**: Writing—review and editing; visualization; writing—original draft; data curation. **Chenxi Yin**: Writing—review and editing; visualization; writing—original draft; data curation. **Keying Li**: Writing—review and editing; visualization; writing—original draft; data curation. **Tian Wang**: Writing—review and editing. **Shiwen Gao**: Writing— review and editing; investigation. **Yue Yu**: Writing—review and editing; investigation. **Cheng Chang**: Writing—review and editing; investigation. **Ye Wang**: Writing—review and editing; investigation. **Guoqing Tong**: Writing—review and editing; investigation. **Liang Gong**: Writing—review and editing; investigation. **Wen Hu**: Writing—review and editing; investigation. **Guoliang Chai**: Writing—review and editing; investigation. **Yilai Han**: Writing—review and editing; investigation. **Qian Qin**: Writing—review and editing; investigation. **Suoqin Jin**: Writing—review and editing; investigation. **Xiufen Zou**: Writing—review and editing; investigation. **Bo Li**: Writing—review and editing; investigation. **Tianyuan Zhang**: Writing—review and editing; visualization. **Ana Conesa**: Investigation; writing—review and editing; visualization; conceptualization.

## CONFLICT OF INTEREST STATEMENT

The authors declare no conflicts of interest.

## ETHICS STATEMENT

No ethics approval was required for this study.

## Supporting information


**Table S1:** Overview of major long‐read sequencing platforms for transcriptomics.
**Table S2:** Overview of long‐read RNA sequencing library strategies and their transcript feature preservation.
**Table S3:** Overview of computational tools for long‐read transcriptomics workflow.

## Data Availability

No new biological data were generated in this review. All code used to generate figures in this paper and render the supplementary website is in the GitHub repository: https://github.com/jia10046/LRS_Doc. Sequencing platform characteristics and typical application scenarios are summarized in Table S1. Customized library preparation strategies are summarized in Table S2. Long‐read transcriptome analysis tools and corresponding recommendations are summarized in Table S3. Supplementary materials (tables, graphical abstract, slides, videos, Chinese translated version, and updated materials) may be found in the online DOI or iMeta Science http://www.imeta.science/.
